# Biocatalytic selective acylation of technical lignins: a new route for the design of new biobased additives for industrial formulations

**DOI:** 10.3389/fchem.2023.1239479

**Published:** 2023-07-21

**Authors:** Aya Sarieddine, Caroline Hadjiefstathiou, Amel Majira, Florian Pion, Paul-Henri Ducrot

**Affiliations:** ^1^ Université Paris-Saclay, Institut national de la recherche agronomique, AgroParisTech, Institut Jean-Pierre Bourgin (IJPB), Versailles, France; ^2^ FARE Laboratory, Institut national de la recherche agronomique, Université de Reims Champagne Ardenne, Reims, France; ^3^ URCOM Laboratory, Université Le Havre Normandie, Le Havre, France

**Keywords:** soda technical lignins, *Candida antarctica* lipase B, selective acylation, lipophilisation, transmethylation

## Abstract

In this article, we describe a proof of concept of the potential use of a biocatalytic process for the functionalization of technical soda lignins from wheat straw through the selective acylation of primary hydroxy groups of lignin oligomers by acetate or hexanoate, thus preserving their free, unreacted phenols. The selectivity and efficiency of the method, although they depend on the structural complexity of the starting material, have been proven on model compounds. Applied to technical lignins, the acylation yield is only moderate, due to structural and chemical features induced by the industrial mode of preparation of the lignins rather than to the lack of efficiency of the method. However, most of the physicochemical properties of the lignins, including their antioxidant potential, are preserved, advocating the potential use of these modified lignins for industrial applications.

## 1 Introduction

Due to the expected gradual depletion of fossil resources, industries have turned to the introduction of polymers, cosmetics, pure natural compounds, or mixtures resulting from the fractionation of the biomass into their formulations. For applications with high added values, the strong consumer demand for environmentally friendly processes and formulations, and a wish for naturalness in commonly used compounds, especially for cosmetics, have strongly stimulated research efforts. Among these compounds and beside secondary metabolites (Vaishnav and Demain, 2011), polysaccharide fractions have received much attention from researchers due to their homogeneity in terms of physicochemical properties and chemical reactivity (Benna-Zayani et al., 2008; Bouyer et al., 2012; Robertson et al., 2017; Arca et al., 2018; Yu et al., 2018). Therefore, industries involved in biomass fractionation have mostly favored “polysaccharides first” biorefinery processes.

**FIGURE 1:**
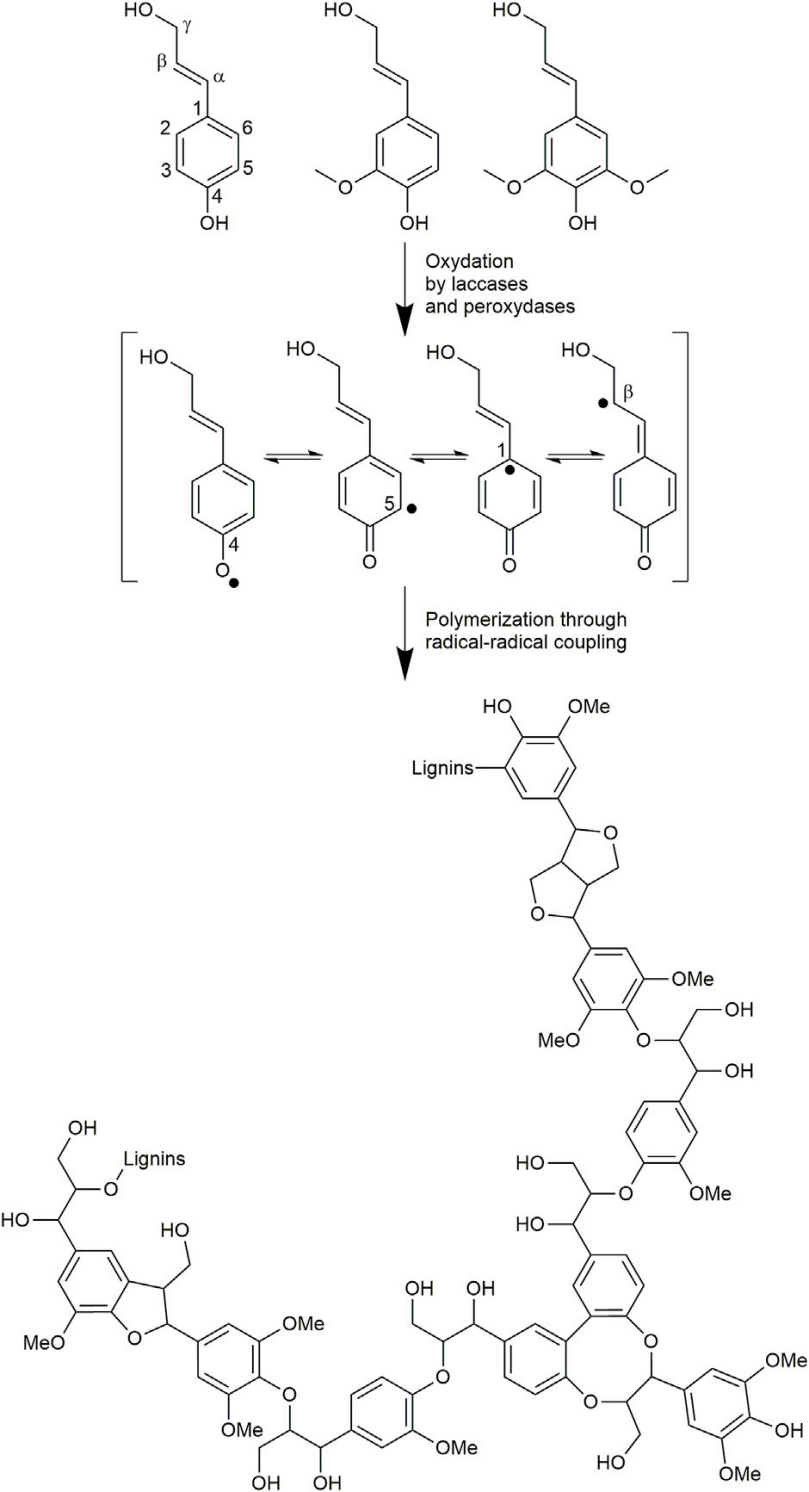
*p*-Hydroxy cinnamic alcohols involved in lignins biosynthesis and putative structure of the resulting biopolymer.

Regrettably, due to the research of optimized polysaccharide or secondary metabolite recovery yields, biomass fractionation processes mostly involve drastic acidic or basic treatments with or without sulfur-containing reagents. Other components of the biomass may be strongly modified structurally, thus highly degrading their chemical structure and their main physicochemical properties of interest. For instance, this is the case with lignins (Sharma et al., 2020; Yao et al., 2022), which represent the major biopolymer in vertical terrestrial plants after cellulose (Ralph et al., 2004). These heterogeneous assemblies of polyphenolic oligomers result in plants from the oxidative coupling of *p*-hydroxy cinnamic alcohols (mainly coniferyl and sinapyl alcohols) and acids (Figure 1) (Liu et al., 2018; Sternberg et al., 2021). Thanks to this unique polyphenolic structure, they exhibit protective properties (such as antioxidant, antimicrobial, and anti-UV) and thus play the role of defensive barriers in plants.

The main treatments of lignocellulosic biomass for polysaccharide recovery and lignin residue availability at an industrial scale for further applications are the Kraft (Chakar and Ragauskas, 2004; Gellerstedt, 2015), bisulfite (Duval et al., 2013; Miles-Barrett et al., 2017), soda (Takada et al., 2020), and organosolv (de la Torre et al., 2013) treatments, which at the end of the process all involve an acidic treatment for the recovery of lignins through precipitation. Thus, large amounts of lignins are produced from paper industries and emerging cellulosic 2G bioethanol industries every year. More than 50 million tons of lignins are produced each year by pulp and paper industries alone (Bezerra and Ragauskas, 2016; Maldhure and Ekhe, 2017; Robertson et al., 2017), and up to now, lignins were considered more as a residue than as a valuable material, and were mainly burned to produce energy because of their high calorific power (Zakzeski et al., 2010; Chen et al., 2017).

In order to complete the biorefinery concept by valorizing all the biomass fractions, the resulting lignins must now be considered as potential candidates for industrial chemical applications. With this aim in mind, low-added value applications such as a filler in asphalt (Wu et al., 2021), additive for mineral wool (Allais et al., 2016; Lucia et al., 2020), and board binders (Gravitis et al., 2010) have emerged. Nevertheless, higher added value applications such as in carbon fibers or as antioxidant additives in polymers and cosmetic formulations have to be explored for technical lignins, which have already shown antioxidant (Pouteau et al., 2003; Vinardell et al., 2008; Ponomarenko et al., 2015; Majira et al., 2019), antimicrobial (Alzagameem et al., 2019; Cresnar et al., 2022), and emulsion stabilizing properties (Czaikoski et al., 2020). For these later applications, incorporation of lignins into most of the usual polymer matrices still faces constraints due to their heterogeneous and complex structure and poor miscibility in apolar matrices, where they show a tendency to form aggregates (Lora and Glasser, 2002; Zhang et al., 2015; Romhányi et al., 2018; Dias et al., 2019). To overcome these problems, it seems that chemical modification of the lignin structure remains the best solution. Degradative depolymerization of lignin has been intensively investigated in past decades (Weng et al., 2021; Roy et al., 2022; Zhou et al., 2022) but requires, whatever the conditions used, final separative processes to recover pure fractions (or, at least, homogeneous in terms of molar mass). Alternatively, another route to minimize their heterogeneity can be solvent fractionation (Majira et al., 2019; Zhou et al., 2022). Some of our previous articles highlighted the potential of phenolic compounds or phenolic fractions derived from lignins constituents as antioxidants (Reano et al., 2016) or as building blocks for polymer chemistry (Pion et al., 2013), and also reported the design of innovative processes for the transformation of technical lignins from various botanical sources and diverse industrial separation processes into valuable fractions (Thierry et al., 2018; Majira et al., 2019; Lu et al., 2021; Jin et al., 2022). In addition, direct modification of lignins to tune their properties may be also a promising pathway, taking into account their valuable chemical functionalities. One example is the acylation of lignin hydroxy groups in order to increase their lipophilicity. Chemical acylation processes in the presence of base catalysts such as 4-dimethyl amino pyridine (DMAP) (Zhao et al., 2017) lead to the formation of byproducts such as carboxylic acids and require organic solvents such as 1,4-dioxane or pyridine. However, most importantly, in such processes, the reactivity of phenolic hydroxyls is higher than that of aliphatic ones, inducing a disappearance of free phenol content and thus a loss of their related properties.

The goal of our project was to target antioxidant additive applications mainly for polymer chemistry; we faced the problem of improving the compatibility of lignins with apolar matrices while not affecting their free phenol content. That is why this project aimed to selectively acylate the primary aliphatic hydroxy groups of some lignin fractions using either acetate or hexanoate groups, thus preserving their antioxidant properties afforded by the presence of the free phenolic groups. We therefore turned to the use of *Candida antarctica* lipase B (CAL-B) as a transesterifying biocatalyst (Cassani et al., 2007; Zhang et al., 2020). This enzyme is indeed known to be inactive toward phenols (Pion et al., 2013; Weissbach et al., 2017) and to be active in a large range of experimental conditions (solvents and temperatures) (Cassani et al., 2007). CAL-B is commercially available in different forms (expression in *Aspergillus niger* (Mustranta et al., 1993) or in yeast (Graber et al., 2003)), either as free enzyme or immobilized on resins (Miletic et al., 2010) in order to allow its easy separation from the reaction mixture by simple filtration. Other groups succeeded with a similar aim of selective acylation of lignins biocatalyzed by CAL-B, involving either ionic liquid as the reaction media (Hulin et al., 2015) or technical lignins that were post-depolymerized through catalytic hydrogenation (Martinez-Garcia et al., 2023). The present work differs in the sense that it involves technical lignin fractions in the solvent MEK, both being industrially common. Moreover, these published works estimated the resulting acylation yields through the disappearance of the acyl donor in the reaction media (by HPLC) or through the decrease of aliphatic hydroxy groups using ^31^P NMR. However, based on our practical experience on lignins analyses and their reactivity, we must consider unpredicted artefacts in such methodologies. Indeed, the lignin spontaneous reactivity that is often noticed in the literature but is not yet fully understood, can generate side-reactions and thus analytical artefacts, leading to misinterpretation. That is why, in this article we furthermore transposed one of the most commonly used method for the quantification of fatty acid composition in complex matrices: transmethylation followed by GC-MS. We follow a robust analytical procedure that is less impacted by the potential biases induced by the unusual chemical reactivity of lignin derivatives upon chemical or thermic treatments. This article describes the feasibility of the combination of a solvent fractionation process and biocatalytic acylation of technical wheat straw alkali lignins (Protobind 1000) to convert them into valuable antioxidant additives for polymers and cosmetic applications as well as a robust analytic methodology to quantify the yield of the acylation step.

## 2 Material and methods

### 2.1 Chemicals

Dimer guaiacylglycerol-β-guaiacyl ether (GGE, Purity >97.0% (GC)) was purchased from TCI Chemicals. Other reagents, laccase (from *Trametes versicolor*, 13 U/mg), and recombinant lipase from *Candida* antarctica supported on acrylic resin (expressed in *Aspergillus niger*, ≥5,000 U/g, (1 U corresponds to the amount of enzyme which liberates 1 µmol of butyric acid per minute at pH 7.5°C and 40°C, from tributyrin as substrate)) were purchased from Sigma Aldrich. Solvents and sulfuric acid (96%) were purchased from Carlo Erba and used as received. Soda technical lignins Protobind 1000 (grass mixture of wheat straw and sarkanda) were purchased from Green Value LLC.

### 2.2 Substrate preparation

#### 2.2.1 Dehydrogenative polymer preparation (DHPs)

S monolignol and syringaresinol were synthesized according to procedure described in one of our previous articles (Jaufurally et al., 2016). 0.4 g of syringaresinol (0.96 mmol) was placed in a 500 mL flask and dissolved in 180 mL of ethanol (EtOH). Then, 200 mL of deionized water was added (1 g/L of total solvents) prior the introduction of 17.6 mg of laccase (0.1 U/mg regarding total substrates). 2 g of S monolignol (9.51 mmol) was dissolved in 20 mL of EtOH (100 g/L) and added dropwise at 0.9 mL/h using a syringe pump. The mixture was stirred in the dark at ambient temperature for 24 h. EtOH was removed under reduced pressure, the mixture was cooled to 0°C, and was then filtered on a glass filter. The same procedure was repeated 10 times and the products gathered in order to obtain a sufficient batch that was dissolved in acetone, then precipitated into diethyl ether. After filtration, the recovered S-DHPs (approximately 10 g) were dried under vacuum.

#### 2.2.2 Fractionation of lignins

Alkali lignins Protobind 1000 (PB1000) was magnetically stirred in ethyl acetate (10 L/kg) for 1 h at room temperature. The mixture was then filtered and flushed with ethyl acetate on a glass filter. The procedure was repeated a second time and the filtrates were combined prior to evaporation under reduced pressure to eliminate ethyl acetate and recover **F1** as a brown solid (36% wt.). The dried insoluble residue was magnetically stirred in methyl ethyl ketone (MEK) (10 L/kg) for 1 h at room temperature. The mixture was then filtered and flushed with MEK on a glass filter. The procedure was repeated a second time and the filtrates were combined prior to evaporation under reduced pressure to eliminate MEK and recover **F2** as a brown solid (26% wt.) and an insoluble residue **F3** (37% wt.).

### 2.3 Procedures for functionalization

#### 2.3.1 Typical procedure for enzymatic acetylation

The substrate to be acetylated (200 mg) was placed in the presence of supported CAL-B (20 mg, 10% mass of the substrate) and mixed with ethyl acetate:acetonitrile (1:1 vol, 20 mL, 10 g/L) in a 25 mL round bottom flask equipped with a Dean–Stark apparatus. The mixture was magnetically stirred under reflux for 24 h and aliquots were periodically collected for kinetics study. After cooling to room temperature, the mixture was filtered to recover the supported lipase and the filtrate was concentrated under reduced pressure to recover the acetylated compound.

#### 2.3.2 DHPs chemical acetylation

S-DHPs (200 mg) was placed in a 25 mL round bottom flask under argon flow in the presence of anhydrous pyridine (2 mL). After cooling to 0°C, acetic anhydride (2 mL, 10 mL/g) was added and the mixture was magnetically stirred at room temperature for 18 h. The reaction was cooled at 0°C before adding methanol dropwise (2 mL). Toluene (2 mL) was then added and the solvents were evaporated under reduced pressure to recover the acetylated DHPs.

#### 2.3.3 Typical procedure for enzymatic hexanoylation

The substrate to be hexanoylated was placed in a round bottom flask in the presence of ethyl hexanoate (1:1 mass ratio to substrate), supported CAL-B (10% mass of substrate), and MEK (25 g/L). The mixture was magnetically stirred under reflux for 24 h using a Dean–Stark apparatus. After cooling to room temperature, the mixture was filtered to recover the supported lipase and the filtrate was concentrated under reduced pressure. In the case of the lignins substrate, the mixture was then precipitated into hexane under magnetic stirring and hexanoylated lignins were recovered by filtration on a glass filter, then dried under vacuum overnight. The parameters that have been changed for the purpose of the study are indicated in the discussion.

### 2.4 Analytical methods

#### 2.4.1 Nuclear magnetic resonance spectroscopy

All NMR spectra were recorded on an Ascend™ 400 spectrometer (Bruker).

##### 2.4.1.1 2D NMR

^1^H-^13^C HMBC and HSQC NMR spectra were recorded using CDCl_3_ (calibrated at 7.26 ppm/77.16 ppm) or pyridine-d_5_ (calibrated at 8.71 ppm/149.9 ppm) as solvent and using standard Topspin 3.2 pulse sequences.

##### 2.4.1.2 31P NMR

Approximately 20 mg of sample and 20 mg of triphenylphosphine (TPP, internal standard) were accurately weighted and dissolved in 0.5 mL of a mixture of CDCl_3_/pyridine (1/1.6) prior to phosphorylation using 2-chloro-4,4,5,5-tetramethyl-1,3,2-dioxaphospholane as the phosphorylating reagent (50 μL). ^31^P spectra were recorded at 160 MHz (128 scans, with a relaxation time of 6 s) and calibrated to the hydrolyzed phosphorylating reagent singlet peak at 132.2 ppm. The following regions were integrated: aliphatic hydroxyls (149.1–144.2 ppm), phenolic hydroxyls (143.8–137.0 ppm), and acid region (136.6–133.6 ppm), normalized to TPP (−5.0 ppm). Each spectrum was treated only when an excess of phosphorylation reagent was observed as a singlet at approximately 175 ppm, and sample analysis was validated if an error below 5% was observed between two duplicates.

#### 2.4.2 Liquid chromatography

Liquid chromatography analyses were performed using a HPLC system (Thermo Fisher Scientific) equipped with an ACCELA 600 pump, an ACCELA auto sampler, a C18 column EC 50/2 Nucleoshell RP 18, 2.7 μm (Macherey-Nagel), and an ACCELA photodiode array (PDA) detector recording over the range 250–450 nm. Samples were dissolved in acetonitrile (ACN) at 1 mg/mL, filtered on a 0.45 µm PET microdisk (Macherey-Nagel), and 1 µL was analyzed using a mobile phase consisting of water/ACN +1‰ HCOOH eluted at 250 μL/min, applying a gradient from 80/20 to 30/70 within 15 min.

#### 2.4.3 Size exclusion chromatography

10 mg of samples was weighed and tetrahydrofuran (THF) containing 5% of toluene (internal standard) was added in order to reach a concentration of 5 mg/mL. After 5 min of shaking, samples were filtered on 0.45 µm GHP microfilters (PALL) prior to analysis by size-exclusion chromatography (SEC). SEC analyses were performed using THF stabilized with BHT as the eluent at 1 mL/min (Ultimate 3000 Pump, Dionex). 10 μL was injected (Ultimate 3000 Autosampler, Dionex) on either a PLgel Mixed C column (5 μm, 7.5 mm × 600 mm, Agilent) in the case of DHPs, or a PLgel Mixed E column (3 μm, 7.5 mm × 600 mm, Agilent) in the case of lignins, and the signal was observed at 280 nm (Ultimate 3000 UV/vis detector, Dionex). The molar mass distributions were determined using a calibration curve based on 10 polystyrene standards ranging from 580 to 364,000 g/mol (Agilent) for the Mixed C column (Supplementary Figure S1) or from 162 to 22,290 g/mol (Agilent) for the Mixed E column (Supplementary Figure S2).

#### 2.4.4 Quantification of hexanoylation yield through transmethylation followed by GC-MS

##### 2.4.4.1 Standard solutions

Different standard solutions have been used in order to allow good accuracy of the calibration curves and an overall good reproducibility of the method. Approximately 500 mg of each internal standard (ethyl pentanoate, methyl heptanoate, methyl octanoate, and tetradecane) was accurately weighed in a 100 mL volumetric flask and mixed with methanol to obtain a stock solution at 5 mg/mL. This stock solution was then diluted by 100 using a 2.5 mL glass syringe and 250 mL volumetric flasks to obtain standard solutions at 0.05 mg/mL in methanol in the presence of 5%vol H_2_SO_4_ (MQS, solution for methyl hexanoate quantification after transmethylation) or not (EQS, solution for residual ethyl hexanoate quantification). In the same manner, a calibration solution (CS) containing methyl hexanoate and ethyl hexanoate, at 5 mg/mL each, in methanol was prepared.

##### 2.4.4.2 Transmethylation procedure

Approximately 20 mg of sample to be analyzed was accurately weighed in a 15 mL Pyrex test tube and 2 mL of internal standard solution containing 5%vol H_2_SO_4_ (**MQS**, 0.05 mg/mL) was added. The capped tube was heated at 80°C for 2 h. After cooling to room temperature, 2 ml of hexane and 1 mL of water were added, the capped tube was agitated vigorously by orbital shaking for 20 min, and then centrifuged at 4°C at 4,800 rpm for 10 min. The upper phase was collected and analyzed directly by GC-MS. Samples were prepared in duplicates and each vial injected twice.

##### 2.4.4.3 Quantification of residual ethyl hexanoate

Approximately 20 mg of sample to be analyzed was accurately weighted in a 15 mL Pyrex test tube and 2 mL of internal standard solution (**EQS**, 0.05 mg/mL) was added. 2 ml of hexane and 1 mL of water were added, the capped tube was agitated vigorously by orbital shaking for 20 min, and then centrifuged at 4°C at 4,800 rpm for 10 min. The upper phase was collected and analyzed directly by GC-MS. Samples were prepared in duplicates and each vial injected twice.

##### 2.4.4.4 Calibration curves

In a 15 mL capped Pyrex test tube, 5–100 µL of the calibration solution **CS** at 5 mg/mL in methanol was added to 2 mL of the internal standard solution **EQS** at 0.05 mg/mL in methanol. 2 ml of hexane and 1 mL of water were added, the capped tube was agitated vigorously by orbital shaking for 20 min, and then centrifuged at 4°C at 4,800 rpm for 10 min. The upper phase was collected and analyzed directly by GC-MS. Each calibration point was prepared in duplicate and injected three times. The vial was validated when an error below 3% was observed between the three injections, and calibration was validated when an error below 5% was observed between the two vials when considering the peak area ratio of compound to quantify over methyl heptanoate. Plotting the determined molar ratio of both compounds to quantify (methyl and ethyl hexanoate) over the methyl heptanoate standard as a function of the corresponding peak area ratios gave their two calibration curves and the corresponding linear equations: [molar ratio (compound/methyl heptanoate)] = f ([peak areas ratio (compound/methyl heptanoate)]) (Supplementary Figure S3).

##### 2.4.4.5 Hexanoylation yield calculation

The chromatogram was validated if 1) the peak area ratio of methyl heptanoate over methyl octanoate was between 0.85 and 0.93 in order to ensure the wellness of the GC-MS system, 2) the peak area ratio of tetradecane over methyl octanoate was between 1.29 and 1.41, in order to ensure the absence of volatiles loss during sample preparation, and 3) in the case of transmethylated samples, no residual ethyl pentanoate was observed in order to ensure complete transmethylation. For a sample to be analyzed, the peak area ratio of the compound to quantify (methyl or ethyl hexanoate) over the methyl heptanoate internal standard was inserted into the equation of its respective calibration curve to determine its concentration. Normalization by sample weight gave its molar amount per Gram of sample to be analyzed. The vial was validated when an error below 5% was observed between the two injections and the sample was validated when an error below 10% was observed between the two vials. The molar amount of residual ethyl hexanoate per Gram of sample was subtracted from the molar amount of methyl hexanoate per Gram of sample quantified after transmethylation to calculate the hexanoylation yield, which was expressed in µmol of hexanoate chains per Gram of sample.

##### 2.4.4.6 Gas chromatography-mass spectrometry procedure

Gas chromatography-mass spectrometry (GC-MS) analyses were performed using a 6890 Series GC system (Agilent). 0.5 µL of sample was injected through a 7683 Series Injector (Agilent) with a split ratio of 30 and the inlet set at 250°C. Elution was by helium N60 at 0.7 mL/min through a DB-Wax UI column (Agilent) with dimensions 30.0 m × 250 μm x 0.25 µm. The oven temperature was maintained at 50°C for 4 min, then increased up to 95°C at 5°C/min, and finally up to 180°C at 70°C/min with a hold of 5 min. Eluted compounds were detected by a quadrupole 5973 Mass Selective Detector (Hewlett Packard) operating over the scanning range 50–650 m*/z*, with electron-impact ionization at 70 eV, the transfer line at 250°C, and the source at 230°C. Identification of compounds was based on pure compound injection and mass spectra comparison. The peak areas of each compound were determined from the total ion chromatogram.

## 3 Results and discussion

### 3.1 Feasibility and selectivity of enzymatic acylation

#### 3.1.1 Acetylation of dimer model compound

**FIGURE 2:**
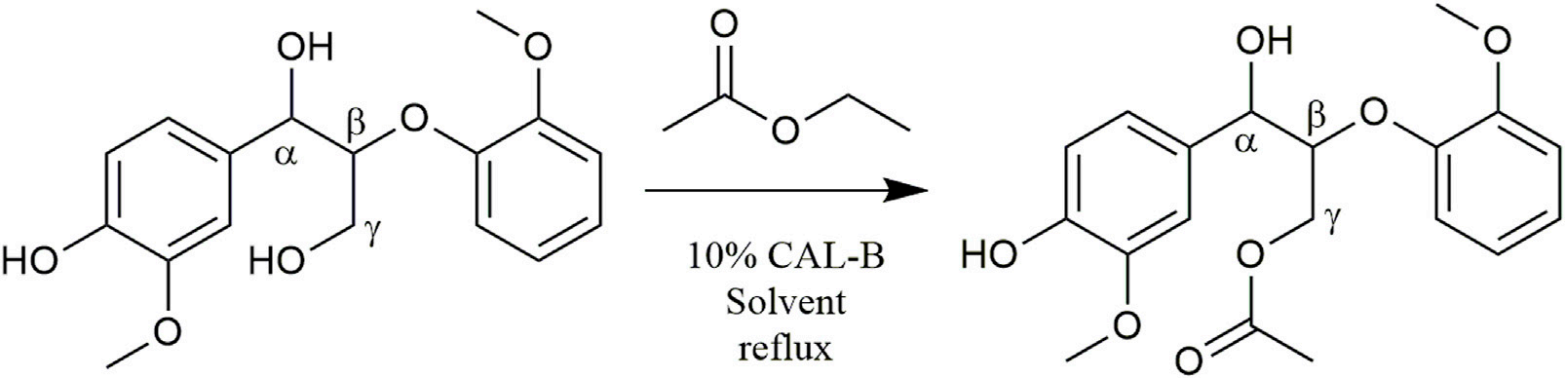
Guaiacylglycerol-β-guaiacyl ether enzymatic acetylation.

**FIGURE 3:**
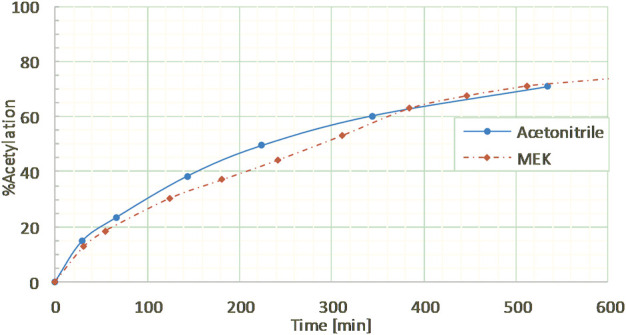
Kinetics of guaiacylglycerol-β-guaiacyl ether (GGE) enzymatic acetylation under reflux in acetonitrile or in MEK (10 g/L), followed by HPLC.

In order to test the feasibility and selectivity of the reaction, the CAL-B activity was first tested on commercial guaiacylglycerol-β-guaiacyl ether dimer, which is commonly used as representative of the β-*O*-4 linkage between two coniferyl alcohols found in lignin oligomers as the major linkage between two *p-*hydroxycinnamic subunits. This commercial stereopure dimer exhibits a free phenolic group and both primary and secondary aliphatic hydroxy groups. The acetylation of the dimer (Figure 2) was first investigated in the presence of ethyl acetate and supported CAL-B (10% wt of dimer) in acetonitrile (10 g/L, 50:50 acetonitrile:ethyl acetate) and with continuous removal of the by-produced ethanol by azeotropic distillation. The reaction was easily followed by HPLC and ^1^H NMR (Supplementary Figure S4). These first results showed only acetylation of the primary hydroxyl in approximately 90% yield after 8 h of reaction. The reaction could also be monitored by ^31^P NMR after phosphorylation (Supplementary Figure S5), where the relative integration of the signal corresponding to the phosphorylated primary OH (triplet at 147.5 ppm) progressively decreased, while the integration of secondary (doublet at 148.2 ppm) and phenolic OH (singlet at 139.5 ppm) remained equal, even if their chemical shift slightly increased (to 148.3 and 139.6 ppm, respectively), thus confirming the selectivity. To further ensure this selectivity, the reaction was conducted over a longer time period (13 days): no peracetylated product was observed by HPLC, ^1^H NMR, or ^31^P NMR. If CAL-B is well-known to be strictly inactive toward phenols, it may induce acylation of secondary hydroxyls (Pion et al., 2013). In this current case, inactivity toward the secondary hydroxyl might be due either to stereoselectivity toward the involved stereopure commercial dimer, or to the proximity of the hindered phenolic moiety (Uppenberg et al., 1995). Acetonitrile, first selected because it is known for being tolerated by CAL-B (Dutta Banik et al., 2016) has however a rather low ability to solubilize lignins. Thus, it was thereafter replaced by methyl ethyl ketone (MEK), showing similar acetylation kinetics on the commercial dimer (Figure 3). MEK was therefore chosen as the reaction solvent for this process and to efficiently solubilize a large fraction of Protobind 1000 (60% wt), which is our targeted lignin substrate.

#### 3.1.2 Complexification of the model substrate: DHPs acetylation

**FIGURE 4:**
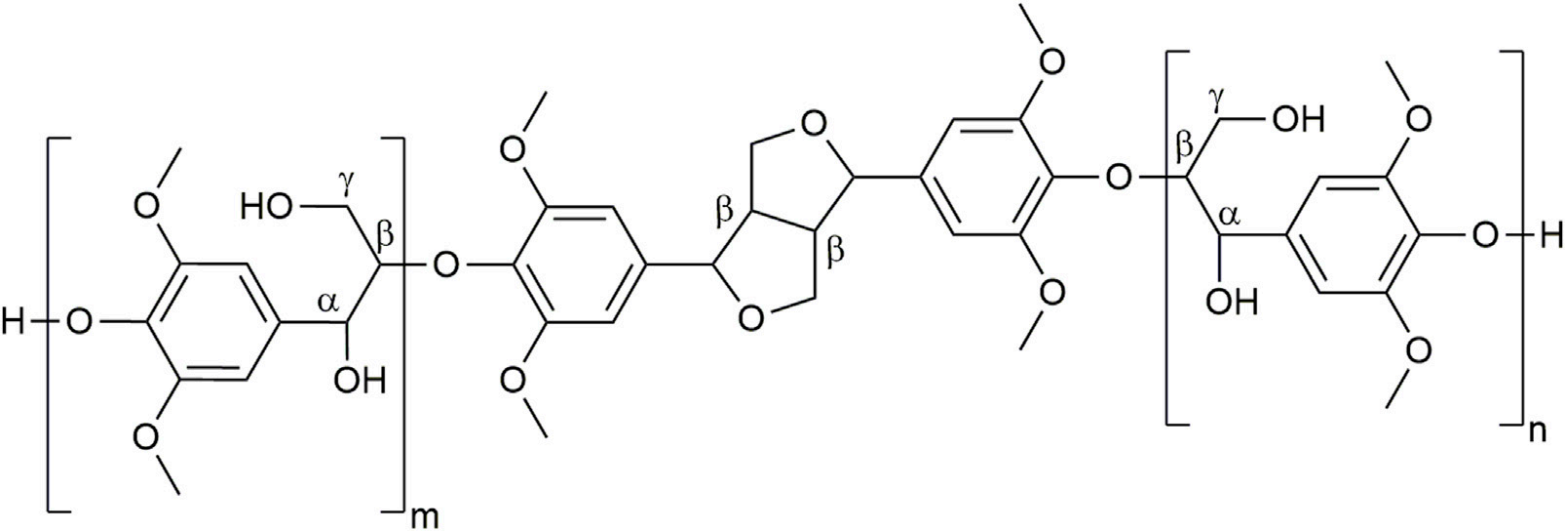
Putative structure of S dehydrogenative polymers (S-DHPs) used as model substrate.

However, direct monitoring of the reaction on lignins fractions by ^1^H NMR or HPLC would be unfeasible due to the complexity of the lignins’ structure and their poor solubility in the appropriate solvents (Wen et al., 2013; Wurzer et al., 2021). Therefore, to challenge further analytical methodologies (SEC, ^31^P NMR), we turned to the acetylation of a substrate of intermediate complexity: dehydrogenative dehydropolymers (DHPs) (Lahive et al., 2020) of sinapyl alcohol (Figure 4). Such S-type DHPs, exhibiting only two types of inter subunit linkages (β-*O*-4 and syringaresinol types), are indeed good candidates to adapt our analytical procedures to a more complex substrate representative of lignins.

DHPs were treated in the presence of CAL-B (10% wt compared to DHPs) in ethyl acetate:MEK (50:50 vol, 10 g/L) under reflux for 2 days. Afterwards, the mixture was cooled to room temperature, filtered to recover supported CAL-B, and the solvent evaporated under vacuum to obtain the resulting acetylated DHPs.

For the purpose of analytical comparisons, completely acetylated DHPs were prepared through a chemical procedure in pyridine using acetic anhydride. From ^1^H-^13^C HMBC NMR (in C_5_D_5_N) of the chemically acetylated DHPs, three acetate groups were observed: one phenolic (2.29 ppm/168.5 ppm) and two aliphatic, corresponding to primary and secondary hydroxyls (1.94 ppm/170.6 ppm and 2.13 ppm/170.0 ppm, respectively), while the enzymatically acetylate showed a single primary aliphatic acetate signal (1.94 ppm/170.6 ppm, Supplementary Figure S6). This confirmed the transposability of the selective enzymatic process to more complex substrates, where acetylation is restricted to primary aliphatic hydroxyls. In addition, another characteristic signal was observed at 2.11 ppm/173.3 ppm, corresponding to residual acetic acid (confirmed by comparison with pure acetic acid, which also appears at 134.6 ppm in ^31^P NMR, Supplementary Figure S7).

**FIGURE 5:**
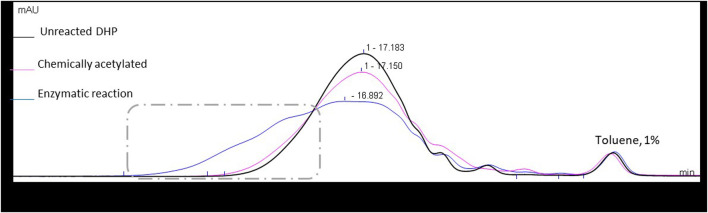
Size exclusion chromatograms in THF of the starting material and the enzymatic and chemically acetylated DHPs, using UV detection at 280 nm. Signals normalized to the toluene peak eluted at 21.1 min.

The molar mass distribution of the samples was also determined through size exclusion chromatography (SEC), assuming that the acetylation could induce an increase in apparent molar mass. Chemically acetylated DHPs showed a slight increase in molar mass distribution when compared to the starting material (Figure 5). Surprisingly, this increase in apparent molar mass was even more significant in the case of enzymatic acetylation, while it should have a lower molar mass gain since its acetylation was partial. Such an observation suggests that side reactions, such as intermolecular cross-coupling reactions, may occur in these reaction conditions in the case of DHPs.

**FIGURE 6:**
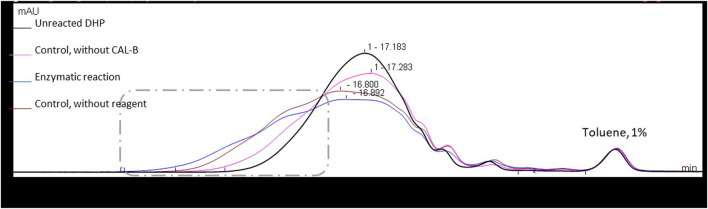
Size exclusion chromatograms in THF for DHPs in different reaction systems (48 h in MEK (10 g/L)), using UV detection at 280 nm. Signals normalized to the toluene peak eluted at 21.1 min.

Therefore, to better understand this unexpected result, two control reactions were run: one in the absence of any acyl donor and another in the absence of the catalyst CAL-B. In the two cases where CAL-B is present (control without acyl donor and enzymatic reaction), a significant increase in molar mass is observed, considering that lipase treatment is able to induce cross-coupling reactions between DHPs residues. In the absence of CAL-B, a similar molar mass increase is observed, but is far less important and probably also due to chemical modifications of the DHP structure upon thermal treatment (Figure 6). The hydroxyl group content of the different samples was then assessed through ^31^P NMR after phosphorylation, a method commonly used for the quantification of the different types of lignin hydroxyls (Table 1) (Meng et al., 2019).

**TABLE 1:** Characteristics of the substrate DHPs (Entry 1), enzymatically acetylated DHPs (Entry 2), and appropriate controls (Entries 3, 4) kept for 48 h under reflux in MEK (10 g/L); molar mass distributions determined ^a^by SEC. Aliphatic and phenolic hydroxyl contents determined ^b^by ^31^P NMR.

Entry	Enzyme (% wt)	Acyl donor	M_n_^a^ (g/mol)	M_w_^a^ (g/mol)	PDI^a^	[Aliphatic OH]^b^ (mmol/g)	[Phenolic OH]^b^ (mmol/g)
1	—	—	1511	2731	1.81	3.32 ± 0.1	1.48 ± 0.05
2	10	Ethyl acetate	1046	3522	3.37	1.98 ± 0.1	1.55 ± 0.07
3	10	—	1101	2733	2.48	2.65 ± 0.05	1.64 ± 0.01
4	0	Ethyl acetate	1109	2676	2.41	2.76 ± 0.02	1.64 ± 0.02
5	10	Ethyl hexanoate	1906	4,658	2.79	2.91 ± 0.04	1.85 ± 0.01

In the case of enzymatic acetylation, the aliphatic hydroxyl content significantly decreased (−40%, Entry 2), suggesting efficient acetylation, as observed by the appearance of an ester spot in ^1^H-^13^C HMBC NMR. Nevertheless, even if lower, this phenomenon was also observed in controls (−20% and −17%, Entries 3 and 4), where no acetate was observed through ^1^H-^13^C HMBC NMR, thus suggesting that this decrease in aliphatic hydroxyls can also be due to spontaneous side reactions. Moreover, this hypothesis is reinforced by an increase in the free phenol content, probably due to the cleavage of some β-*O*-4 bonds inside the oligomers. From such global observations it can be assumed once more that DHPs (and probably also lignin fractions) may undergo dramatic structural changes in these reaction conditions (temperature, solvent), including for instance β-*O*-4 bond cleavage and recondensation reactions. The balance between these two pathways, going from control reactions to the real reaction may be to some extent due to the generation of acetic acid in the medium containing the ethyl acetate and CAL-B, but also to the specific activity of CAL-B on lignin oligomers in the absence of any acyl donor.

Although confirming the occurrence of side reactions impacting the hydroxyl content in all cases, ^31^P NMR appeared to be nevertheless unsuitable for precise quantification of the acylation yield for DHPs (and probably for lignin fractions), contrary to our expectations. Indeed, the acetylation yield, when estimated on the basis of ^31^P NMR data, seemed largely overestimated (approximately 50% mol of the putative primary hydroxy groups). We thus concluded that another analytical method was needed to quantify the acylation yield. We envisaged the use of an indirect method based on either saponification or transesterification methods, allowing the reformation of acids or esters from the acylated lignins, which can be further quantified by GC-MS. However, such a process is incompatible with acetylated compounds since it generates either acetic acid or methyl acetate, which are difficult to quantify by GC-MS. Another longer acyl donor was therefore chosen, ethyl hexanoate, as its excess can still be easily removed by either evaporation or hexane washing, and since its corresponding acid or methyl ester are easily quantifiable by GC-MS.

#### 3.1.3 Hexanoylation of the dimer model compound

**FIGURE 7:**
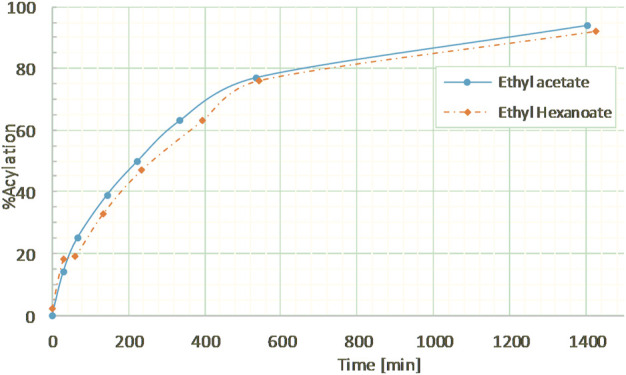
Kinetics of guaiacylglycerol-β-guaiacyl ether acylation by ethyl acetate or ethyl hexanoate, in MEK (10 g/L) under reflux, followed by ^1^H NMR in CDCl_3_.

The hexanoylation process was first tested on the commercial dimer in order to assess the efficiency of this approach with a longer chain and a more apolar acyl donor. Guaiacylglycerol-β-guaiacyl ether dimer was thus reacted with ethyl hexanoate in a ratio of 5:1 mol to ensure an excess of acyl donor at the same conditions (10% wt CAL-B, MEK, 10 g/L, reflux). The kinetics of hexanoylation was monitored by ^1^H NMR (Supplementary Figure S8) and HPLC and was found to be very similar as the acetylation (Figure 7), with a hexanoylation yield of 76% after 9 h, thereby proving the versatility of the method in terms of acyl donor. Similar control experiment ran without CAL-B induced no structural changes of guaiacylglycerol-β-guaiacyl ether dimer (results not shown), thus confirming that hexanoylation was indeed only catalyzed by CAL-B.

#### 3.1.4 Hexanoylation of DHPs and quantification of the acylation yield by a transmethylation/GC-MS procedure

DHPs were then reacted with an excess of ethyl hexanoate (5:1 wt) in MEK (10 g/L) under reflux. After the biocatalyzed hexanoylation reaction and the removal of the supported CAL-B by filtration, the reaction medium was concentrated under reduced pressure and first cleaned from residual unreacted acyl donor through precipitation in hexane, and submitted to extended drying under vacuum. ^1^H-^13^C HMBC NMR proved the formation of an ester bond (Supplementary Figure S9) as well as the absence of residual acyl donor (no ethyl spots visible), which was confirmed by GC-MS analysis of the hexane supernatant. Saponification of the hexanoylated compounds was first tested but led to very tedious workup procedures and unsatisfying errors in the quantification of the resulting hexanoic acid. We thus turned to a transmethylation process, well-known in the field of triglyceride chemistry. Transmethylation aims to cleave the ester bonds and thus release the hexanoate moieties as methyl esters (Supplementary Figure S10). Thereafter, transmethylation followed by GC quantification established that 184 µmol of methyl hexanoate was released per Gram of reacted DHPs. This value, compared to the aliphatic hydroxyl content of the starting material determined by ^31^P NMR (3.32 mmol/g, Table 1 Entry 5), indicates a hexanoylation yield of 5.5% mol of the aliphatic hydroxy groups; nevertheless, such a calculation underestimates the yield as it does not take into account the occurrence of side reactions decreasing the amount of available targeted aliphatic hydroxy groups and does not discriminate primary from secondary hydroxyl content, which may be equivalent according to the putative DHPs structure. Structural modification of DHPs was not investigated further as it was not the aim of this work; however, it appears more reliable to quantify acylation through transmethylation rather than structural analysis (^31^P NMR or SEC), as we expected initially. For these reasons, the hexanoylation yield will be estimated by transmethylation and expressed in µmol/g. In initial attempts, we compared the quantification through peak area measurement inserted in the equation [molar concentration] = f ([peak area]) determined by injection of **CS** at different concentrations. Nevertheless, the use of an internal standard of close structure (methyl heptanoate, **MQS**) led to higher repeatability and was preferred in the remainder of our study, as described in the material and methods section.

This method is indirect and it can be applied to any acylated substrate of complex structure, as far as the residual unreacted acyl donor is quantified or, when possible, carefully eliminated. Therefore, this method was retained for hexanoylation yield estimation in the remainder of the study, aiming to transfer this biocatalyzed selective hexanoylation process to technical lignins and derived fractions.

### 3.2 Transposition of the enzymatic hexanoylation to lignins

#### 3.2.1 Fractionation of the technical lignins

**FIGURE 8:**
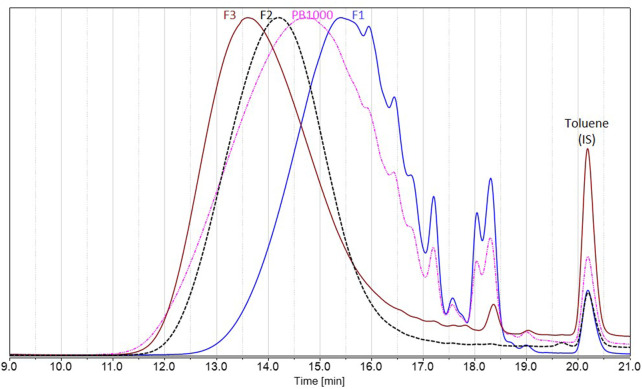
Size exclusion chromatography in THF of Protobind 1000 technical lignins (pink) and the resulting fractions F1 (blue), F2 (black), and F3 (brown), using UV detection at 280 nm. Signals normalized to the maximum height; toluene used as internal standard, eluted at 20.2 min.

To deal with less heterogeneous samples than the complex technical lignins Protobind 1000, sequential solvent fractionation was applied in order to obtain more defined lignin fractions (Lu et al., 2021; Jin et al., 2022). The first step involved ethyl acetate in order to eliminate lower molar mass compounds **F1** and to focus on polymeric chains, as they encounter more difficulties in accessing enzyme active sites. The second step involved MEK in order to extract a substrate soluble **F2** in the reaction media (MEK), as well as in analytical solvents, by eliminating the insoluble residue **F3**. These three fractions showed lower polydispersities than Protobind 1000 and exhibited increasing molar masses as follows: M_w_ (**F1**) < M_w_ (Protobind 1000) < M_w_ (**F2**) < M_w_ (**F3**), while the phenol content decreased in the opposite manner (Figure 8; Table 2).

**TABLE 2:** Characteristics of the different fractions (ethyl acetate F1, MEK F2, and insoluble F3 fractions) obtained from Protobind 1000 (PB1000) determined ^a^by gravimetry, ^b^by SEC, and ^c^by ^31^P NMR (spectra available in Supplementary Materials).

	%wt^a^	M_n_^b^ (g/mol)	M_w_^b^ (g/mol)	PDI^b^	[Aliphatic OH]^c^ (mmol/g)	[Phenolic OH]^c^ (mmol/g)	[COOH]^c^ (mmol/g)
PB1000	100	573	1744	3.04	1.94 ± 0.02	4.43 ± 0.05	1.17 ± 0.03
**F1**	36	385	813	2.11	1.16 ± 0.04	5.24 ± 0.09	1.27 ± 0.01
**F2**	27	1240	2188	1.76	1.41 ± 0.01	4.13 ± 0.01	1.02 ± 0.02
**F3**	37	1266	2695	2.13	2.50 ± 0.07	3.34 ± 0.12	0.96 ± 0.05

#### 3.2.2 Hexanoylation of lignins fraction F2

The hexanoylation of soda lignins fraction **F2** (lignins fraction soluble in MEK but insoluble in ethyl acetate) was thus conducted in MEK with ethyl hexanoate. The first attempt was conducted based on previous experimental parameters: 5 mass equivalent of ethyl hexanoate and 10% wt CAL-B regarding lignins were used in MEK (10 g/L) under reflux for 24 h, which led to a hexanoylation yield of 85 μmol/g. The same experiment conducted in absence of CAL-B showed no hexanoylation; this control informed us that CAL-B activity was responsible for the reaction and confirmed the accuracy of hexanoylation yield quantification through transmethylation followed by GC-MS, inducing no overestimation. In order better understand the reactivity of this new system, different key parameters have been studied to optimize enzymatic hexanoylation: 1) ethyl hexanoate:lignins weight ratio, 2) reaction time, 3) lignins concentration, 4) enzyme load, and 5) the solvent used as the reaction medium. In all following sections, different assays were compared based on their resulting hexanoylation yields determined through transmethylation followed by GC-MS. Some of the following studies were conducted in parallel; therefore, the fixed parameters are not necessarily optimized, aiming to detect tendencies rather than defining an optimized process.

##### 3.2.2.1 Ethyl hexanoate:lignins weight ratio

The work on model DHPs was conducted with 5 mass equivalent of ethyl hexanoate in order to ensure a sufficient excess. However, such an excess led to tedious removal of residual unreacted acyl donor at the end of the process. Thus, various ethyl hexanoate:lignins weight ratios (from 0.05 to 5 mass equivalents) were studied at a constant enzyme load (20% wt), lignins concentration in MEK (10 g/L), and reaction time (24 h). Lowering the weight ratio of ethyl hexanoate:lignins from 5:1 to 1:1 gave similar results (102 and 94 μmol/g, respectively), while too low a ratio led to a drop in the hexanoylation yield (25 μmol/g with 0.05:1, Figure 9).

**FIGURE 9:**
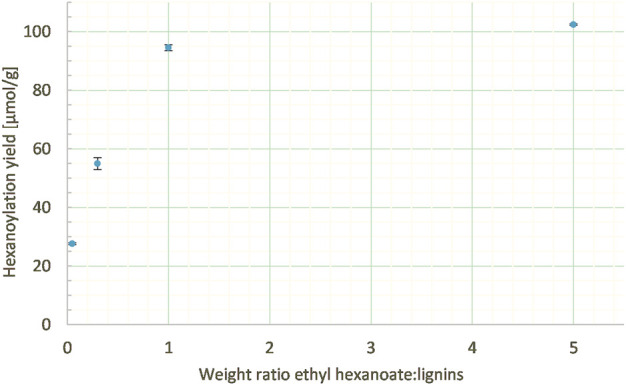
Hexanoylation yield obtained for different ethyl hexanoate:lignins weight ratios at a constant enzyme load (20% wt), lignins concentration in MEK (10 g/L), and reaction time (24 h); yield estimated by transmethylation followed by GC-MS.

Thus, a 1:1 weight ratio was preferred as it makes the post-reaction workup easier and the process cheaper and greener by generating less side-products without significantly decreasing the hexanoylation yield.

##### 3.2.2.2 Reaction time

**FIGURE 10:**
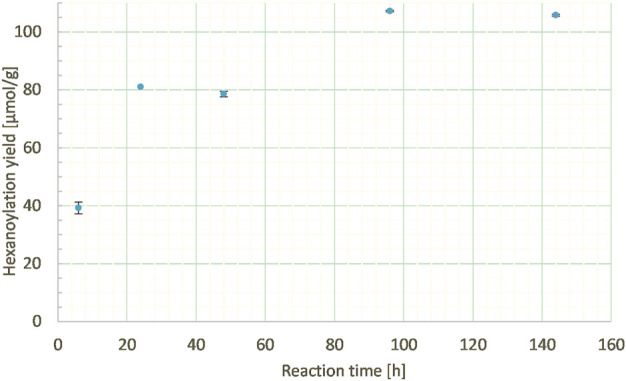
Hexanoylation yield obtained for different reaction times at a constant ethyl hexanoate:lignins weight ratio (1:1), enzyme load (10% wt), and lignins concentration in MEK (10 g/L); yield estimated by transmethylation followed by GC-MS.

Another key parameter can be the reaction time; thus, it was studied from 6 h to 6 days at a constant ethyl hexanoate:lignins weight ratio (1:1), enzyme load (10% wt), and lignins concentration in MEK (10 g/L). 6 h reaction time led to only half of the hexanoylation yield (39 μmol/g) compared to 24 h (81 μmol/g), indicating an incomplete process. Further extension of the reaction time led to a slight increase in the hexanoylation yield (Figure 10). Thus, time can be a parameter of interest but should be balanced according to the energetic cost and lignins side reactions.

##### 3.2.2.3 Lignins concentration in MEK

**FIGURE 11:**
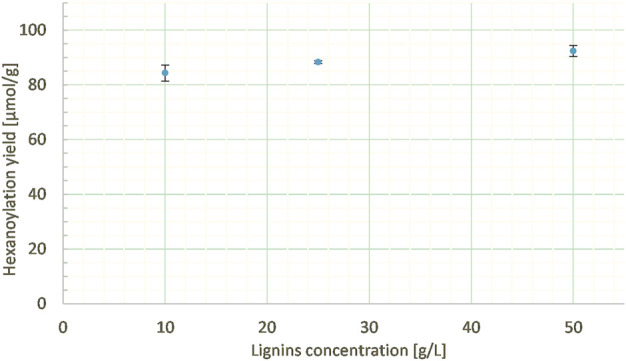
Hexanoylation yield obtained for different reaction concentrations at a constant ethyl hexanoate:lignins weight ratio (5:1), enzyme load (10% wt), solvent nature (MEK), and reaction time (24 h); yield estimated by transmethylation followed by GC-MS.

The lignin concentration in MEK was varied from 10 to 50 mg/mL at a constant ethyl hexanoate:lignins weight ratio (5:1), percentage of enzyme (10% wt), and reaction duration (24 h). Similar hexanoylation yields (from 85 to 92 μmol/g) were obtained in the three cases (Figure 11), demonstrating the low impact of concentration on the studied range, thus allowing us to reduce the amount of solvent employed.

##### 3.2.2.4 CAL-B load

**FIGURE 12:**
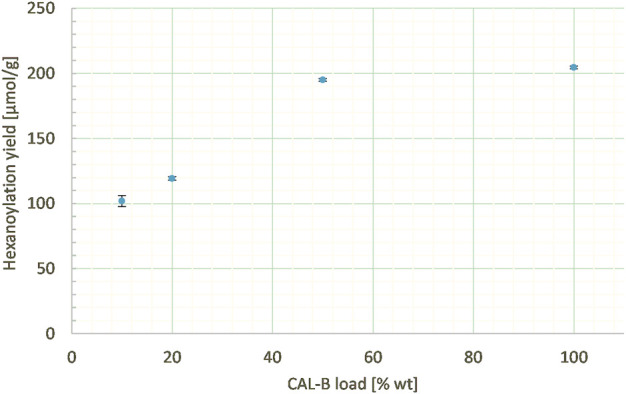
Hexanoylation yield obtained for different CAL-B loads at a constant ethyl hexanoate:lignins weight ratio (1:1), lignins concentration in MEK (25 g/L), and reaction time (24 h); yield estimated by transmethylation followed by GC-MS.

In order to assess the impact of catalyst load on the reactivity, the percentage of supported enzyme was also varied from 10% to 100% wt according to lignins at a constant ethyl hexanoate:lignins weight ratio (1:1), lignins concentration in MEK (25 g/L), and reaction time (24 h). Indeed, the hexanoylation yield increased along with the CAL-B load (Figure 12), even if the phenomenon is far from being linear: increasing the load by 10 times doubled the yield (250 μmol/g). Thus, the CAL-B load is a real lever for increasing the hexanoylation yield; nevertheless, due to its high cost, a balance should be struck between cost and efficiency.

##### 3.2.2.5 Solvent variation

**FIGURE 13:**
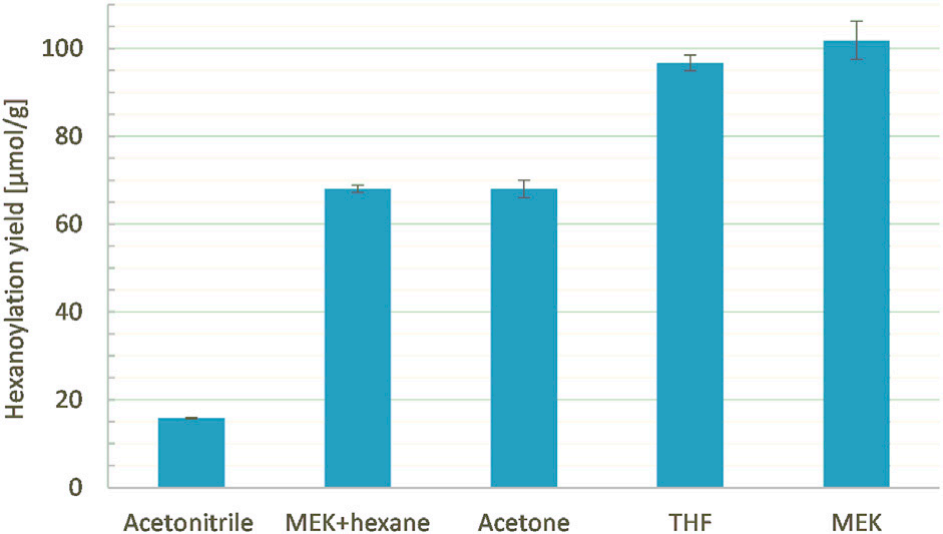
Hexanoylation yield obtained for different solvents as reaction media at a constant ethyl hexanoate:lignins weight ratio (1:1), enzyme load (10% wt), lignins concentration (25 g/L), and reaction time (24 h); yield estimated by transmethylation followed by GC-MS.

It is known that CAL-B activity and stability are impacted by the polarity and temperature of the medium (Kitamoto et al., 2015; Banik et al., 2016). In our case, both are governed by the solvent chosen, which also impacts substrate solubilization. Thus, solvents of different polarities and different boiling points (acetonitrile, acetone, MEK, THF, or a mixture MEK/hexane (50/50), Table 3) were tested at a constant ethyl hexanoate:lignins weight ratio (1:1), enzyme load (10% wt), lignins concentration (25 g/L), and reaction time (24 h, Figure 13). Acetone, which shows a polarity similar to MEK (Hidebrand parameters of 19.9 and 19.3 MPa^1/2^, respectively) but a lower boiling point (56°C and 80°C, respectively), led to a lower hexanoylation yield (68 μmol/g instead of 102 μmol/g). This lower reactivity might be related to the lower temperature, which decreases the CAL-B activity and/or impeaches ethanol removal. Acetonitrile, known to be compatible with CAL-B (Arcens et al., 2020) and suitable as a dimer acetylation medium, exhibits a boiling point (82°C) similar to MEK, but a higher Hildebrand parameter (24.3 MPa^1/2^); the significantly lower hexanoylation yield (16 μmol/g) when compared to MEK might be due to the low solubility of lignins **F2** in acetonitrile, where only 65% was recovered (generally 90% is recovered). THF shows a similar Hildebrand parameter (18.6 MPa^1/2^) but a lower boiling point than MEK (66 °C), leading to a similar hexanoylation yield of 97 μmol/g. THF appears therefore to be a choice for substrates of lower solubility. Finally, a mixture of MEK and hexane (50/50) was attempted in order to improve ethanol removal. The resulting hexanoylation yield was only 68 μmol/g, most likely due to the lower solubility of the substrate **F2** in this system (only 72% recovered). These overall observations tend to indicate that MEK is a good solvent of choice in our system. When one wants to change for another solvent, attention should be paid to both its boiling point (60°C–100°C) and its polarity (19–20 MPa^1/2^) in order to preserve a certain reactivity.

**TABLE 3:** Characteristics of the tested solvents and the resulting recovery (after precipitation in hexane) and acylation yields.

Solvent	Hildebrand parameter (MPa^1/2^)	Boiling point (°C)	Recovery yield (%)	Acylation yield (µmol/g)
Acetone	**19.9**	**56**	**90**	**68**
Acetonitrile	**24.3**	**82**	**65**	**16**
MEK	**19.3**	**80**	**89**	**102**
MEK/Hexane (50/50)	**nd**	65	**72**	**68**
THF	**18.6**	**66**	**90**	**97**

## 4 Conclusion

This study demonstrates the feasibility of the enzymatic selective acylation of primary hydroxy groups of lignin fractions, using, first, well controlled model compounds (commercial guaiacylglycerol-β-guaiacyl ether and sinapyl alcohol dehydrogenative polymers) in order to design and ensure reproducible efficiency of the analytical protocols to be used for the estimation of the acylation yield, as well as the selectivity of the process strictly inactive toward phenols. The whole process, applied to fractions of commercial wheat straw soda lignins Protobind 1000, led to relatively moderate hexanoylation yields (approximately 100 μmol/g), much lower than those obtained with model compounds. This drastic yield decrease is not only a result of the more complex structure of lignin fractions compared to DHPs, but also the process used for the preparation of the starting lignins (remaining salts and low pH of the resulting lignin fraction solutions, which may favor critical changes in the structure of the lignins). From a methodological point of view, we have also demonstrated that the transmethylation method is a versatile method for the determination of the acylation yield, more so than the direct chromatographic (SEC) and spectroscopic (^31^P NMR) methods, the former not allowing direct quantification and the latter leading to a large overestimation of the acylation yield due to side-reactions, as demonstrated with DHPs. Moreover, this analytical approach involving transmethylation can be applied to any substrate, regardless of its structural complexity or solubility. Different hexanoylation conditions can be considered (in terms of solvent type, concentration, duration, and CAL-B load); thus, they can be adapted regarding 1) substrate specificities if required and/or 2) cost/efficiency balance target. Different experimental conditions have been tested with modified parameters and how they impact the acylation yield, allowing the design of a large panel of procedures that can be used according to the nature of the substrate (solubility and complexity) and the target efficiency. In future, we will diversify the acyl donor as well as the substrates in order to investigate the influence of biomass botanical origin and the impact of the lignin recovery procedure on the reactivity, assuming that the yield of this acylation reaction may be one of the possible quality criteria for technical lignins for further possible valorization routes. It is important to note that our preliminary results concerning the use of these modified lignins as either antioxidant additives for polymer compounding or as stabilizers for cosmetic emulsions are quite promising and demonstrate the significant advantage of modified lignins compared to starting lignins. These different studies are underway and will be reported in due time.

## Data Availability

The original contributions presented in the study are included in the article/Supplementary Materials, further inquiries can be directed to the corresponding author.
