# Supplementary material for: Biocatalytic selective acylation of technical lignins: a new route for the design of new biobased additives for industrial formulations
Source: Front Chem. 2023 Jul 21;11:1239479. doi: 10.3389/fchem.2023.1239479 (PMC10400768; doi:10.3389/fchem.2023.1239479)
Supplement: Supplementary file 1 [file DataSheet1.PDF]

Supplementary Material

Biocatalytic selective acylation of technical lignins: a new route for the design of new biobased antioxidant additives for industrial formulations

Aya Sarieddine, Caroline Hadjiefstathiou, Amel Majira, Florian Pion and Paul-Henri Ducrot*

*** Correspondence:** Paul-Henri Ducrot: paul-henri.ducrot@inrae.fr

1 Supplementary Figures and Tables

1.1 Supplementary Figures

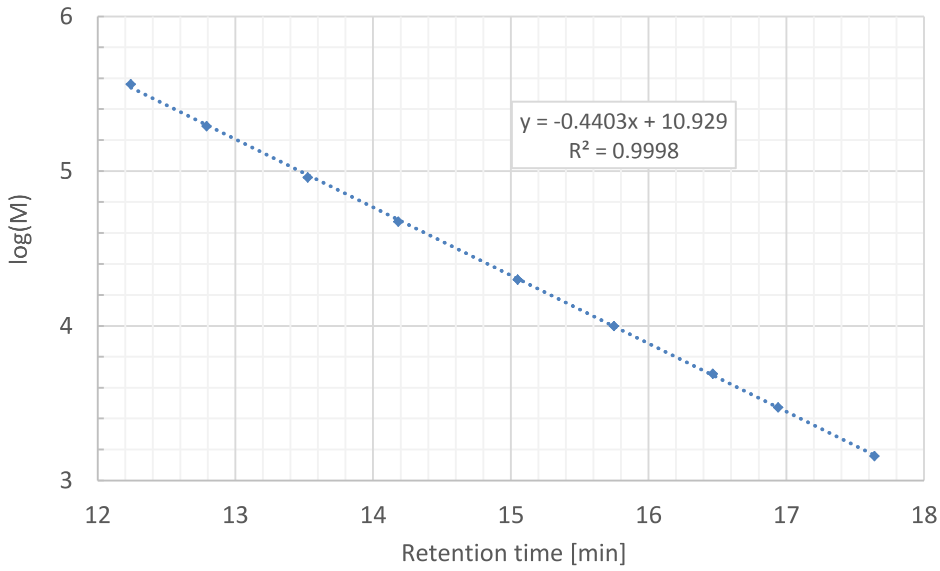

Supplementary Figure 1. SEC calibration curve obtained from 9 polystyrenes standards (from 580 to 364,000 g/mol) in THF at 1 mL/min using a Mixed C column.

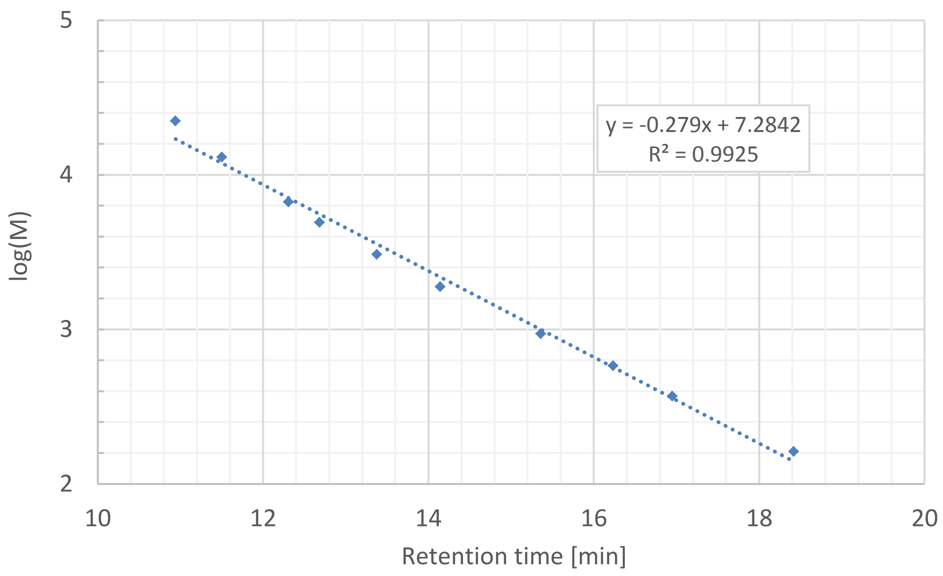

Supplementary Figure 2. SEC calibration curve obtained from polystyrenes standards (from 162 to 22,290 g/mol) in THF at 1 mL/min using a Mixed E column.

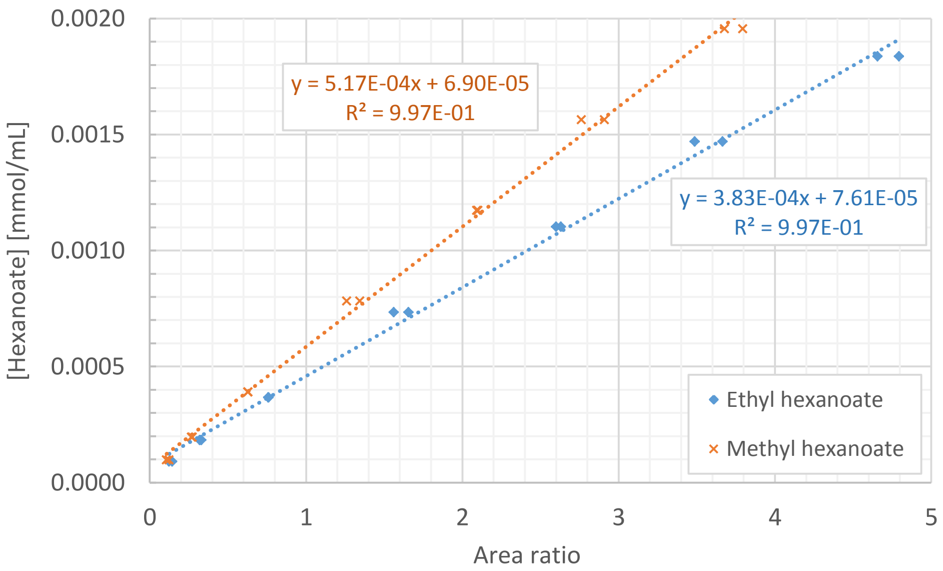

Supplementary Figure 3. Calibration curves for quantification by GC-MS of residual unreacted ethyl hexanoate (in blue) and methyl hexanoate (in orange) released by transmethylation using EQS and MQS reference solutions; areas of compound to calibrate were divided by methyl heptanoate area.

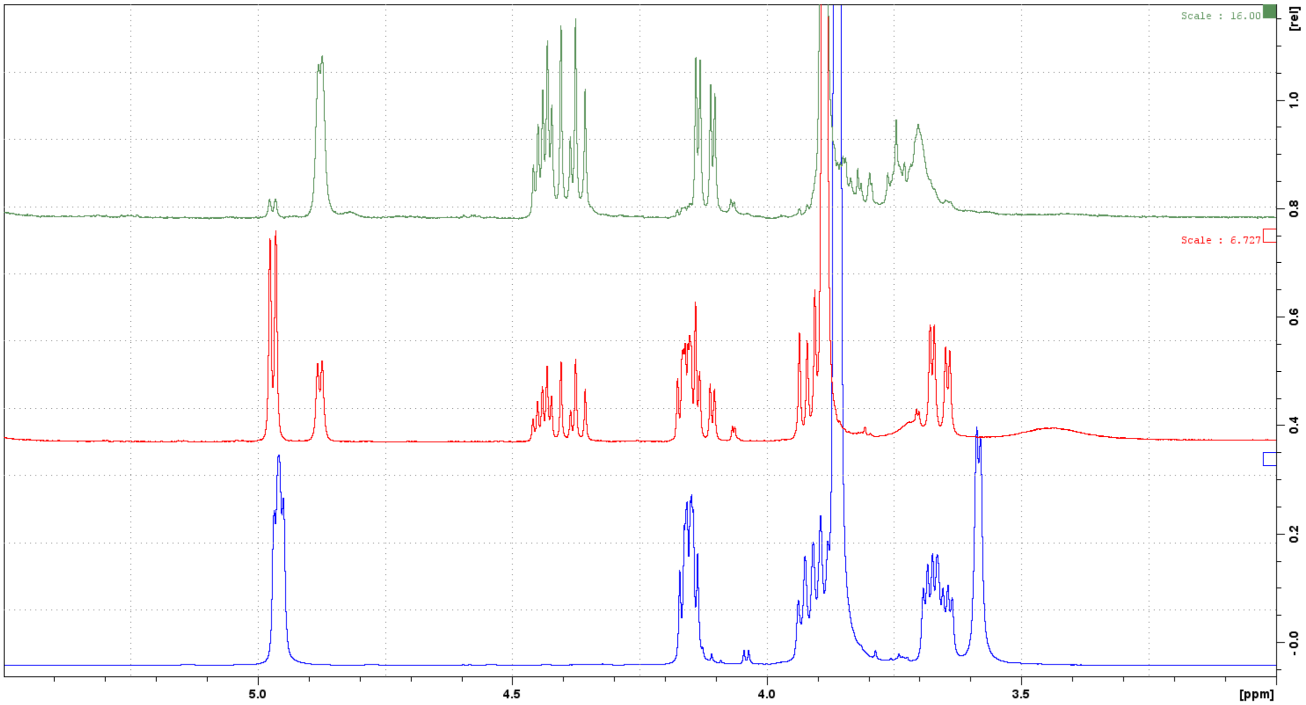

Supplementary Figure 4. Kinetic of guaiacylglycerol-β-guaiacyl ether enzymatic acetylation followed by ^{1}H NMR at t = 0 min (blue), after 144 min (red) and 1404 min (green); aliquots of 500 µL were periodically collected, dried under vacuum and dissolved in $CDCl_3$; spectra were calibrated on $CDCl_3$ at 7.26 ppm; zoom on 5.5-3.0 ppm; %Acetylation = 100 x Area(4.89 ppm)/Area(4.98 + 4.89 ppm)

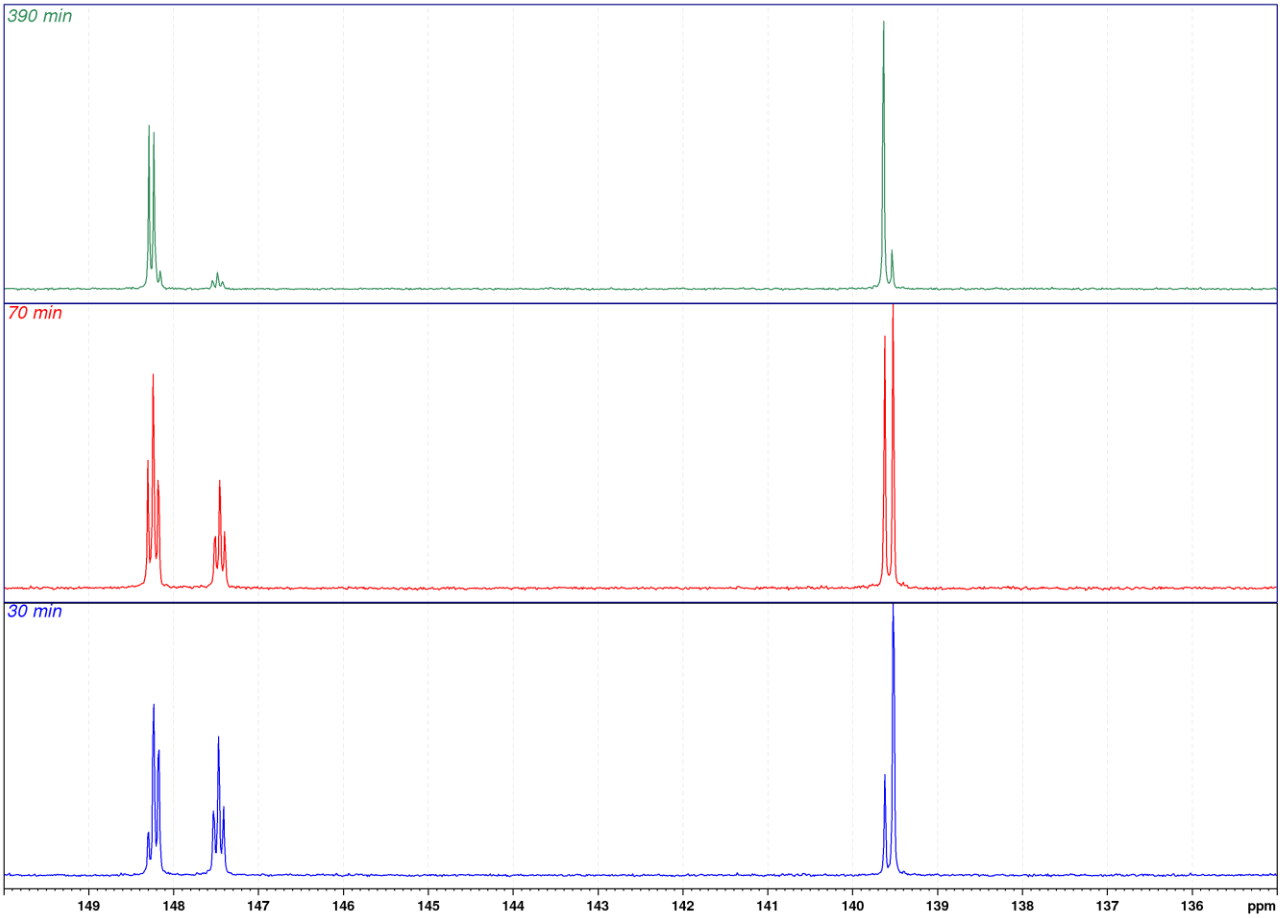

Supplementary Figure 5. Kinetic of guaiacylglycerol-β-guaiacyl ether enzymatic acetylation followed by ^{31}P NMR after phosphorylation after 30 min (blue), 70 min (red) and 390 min (green); aliquots of 500 µL were periodically collected, dried under vacuum and dissolved in pyridine/$CDCl_3$ prior phosphorylation; chemical shift of the spectra were calibrated on hydrolyzed phosphorylating reagent at 132.2 ppm for the chemical shifts, and integration on the peak at 148.3 ppm as 1; zoom on 150-135 ppm; %Acetylation = 100 x (1 - Area(147.5 ppm))/Area(148.3 ppm).

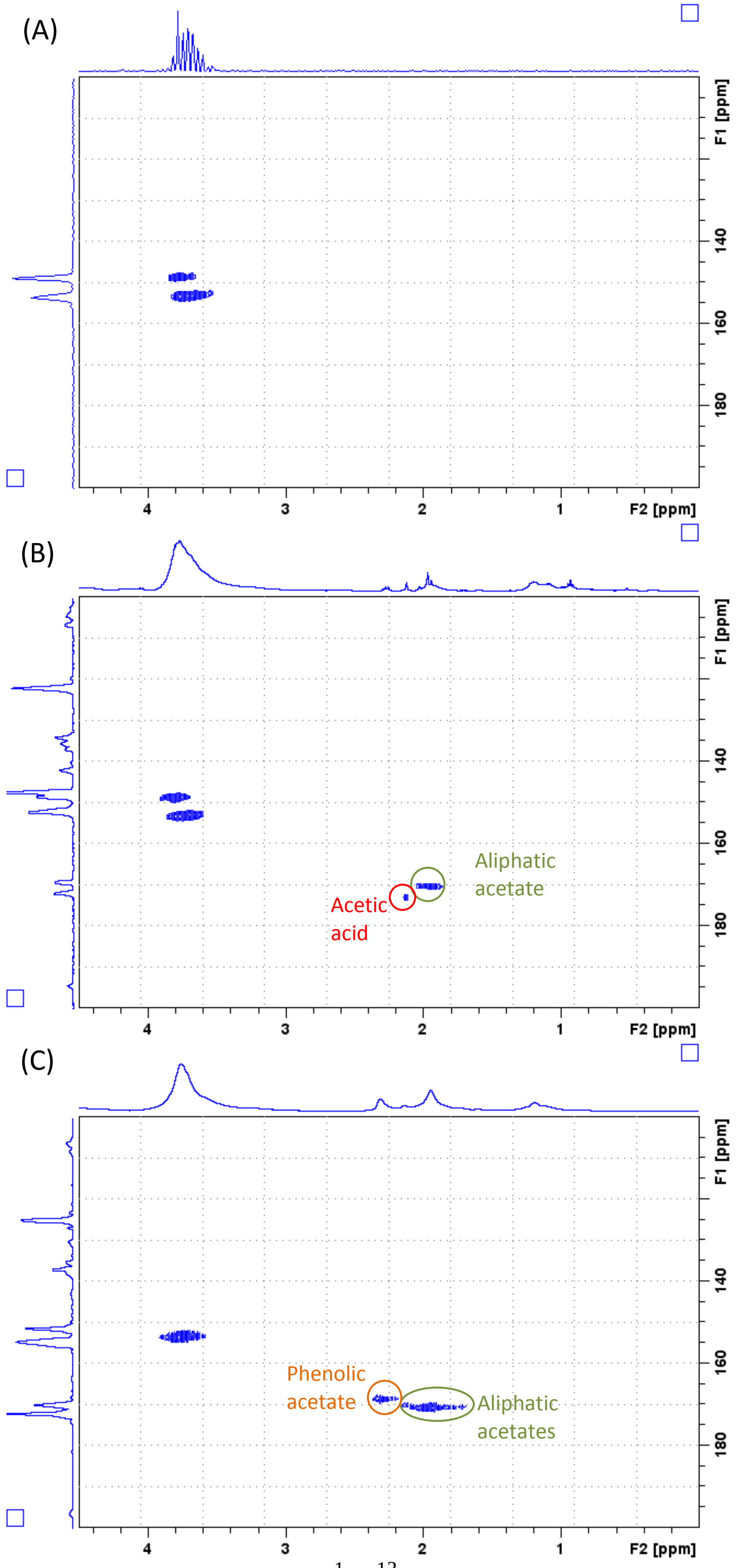

Supplementary Figure 6. ^{1}H-^{13}C HMBC NMR spectra in pyridine-d5 of (A) the starting material (DHPs), (B) enzymatically acetylated DHPs and (C) chemically acetylated DHPs; zoom on 4.5-0/200-100 ppm.

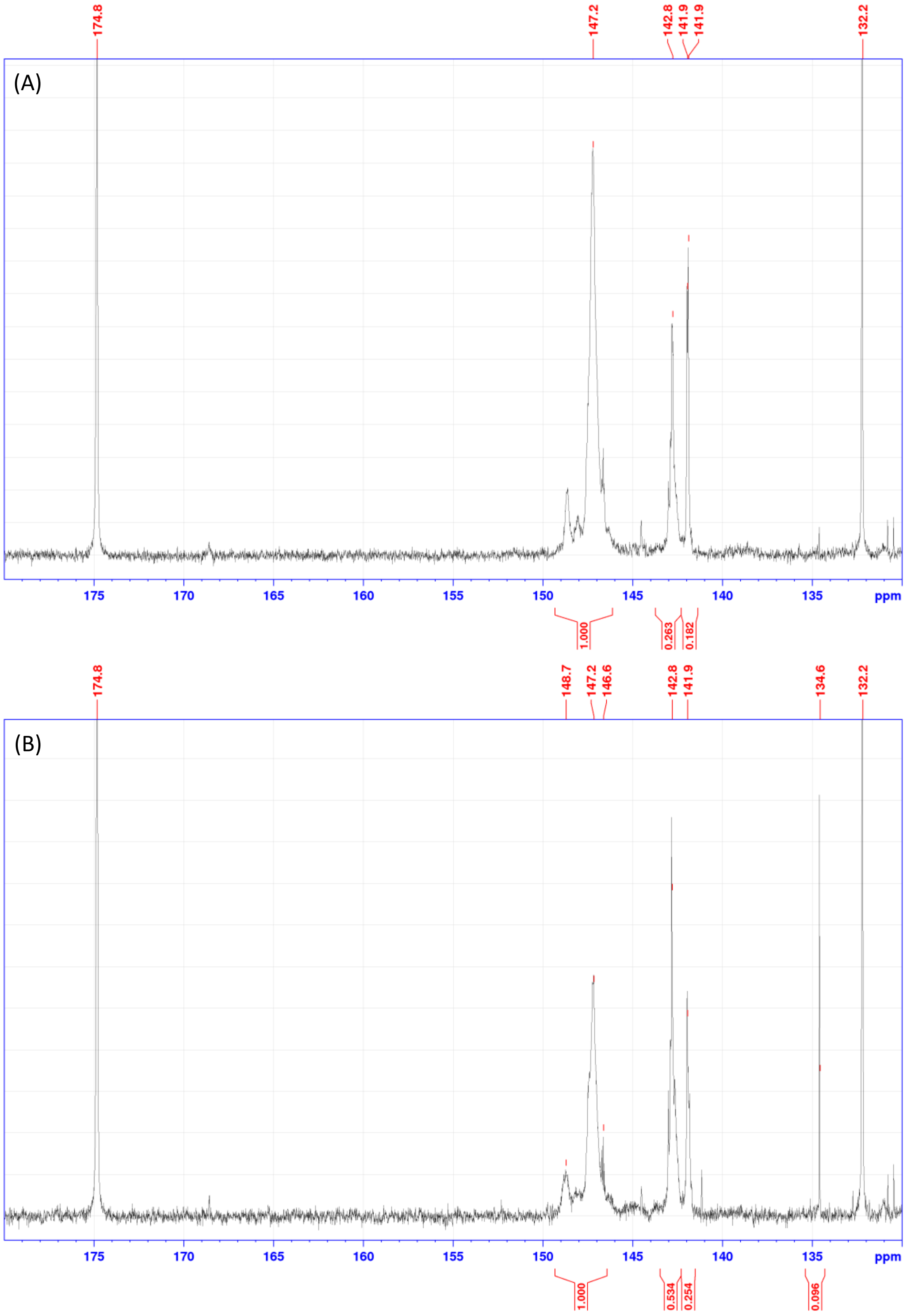

Supplementary Figure 7. ^{31}P NMR spectrum of (A) S-DHPs substrate and (B) enzymatically acetylated DHPs after phosphorylation in pyridine/$CDCl_3$; spectrum calibrated on hydrolyzed phosphorylating reagent at 132.2 ppm; zoom on 180-130 ppm.

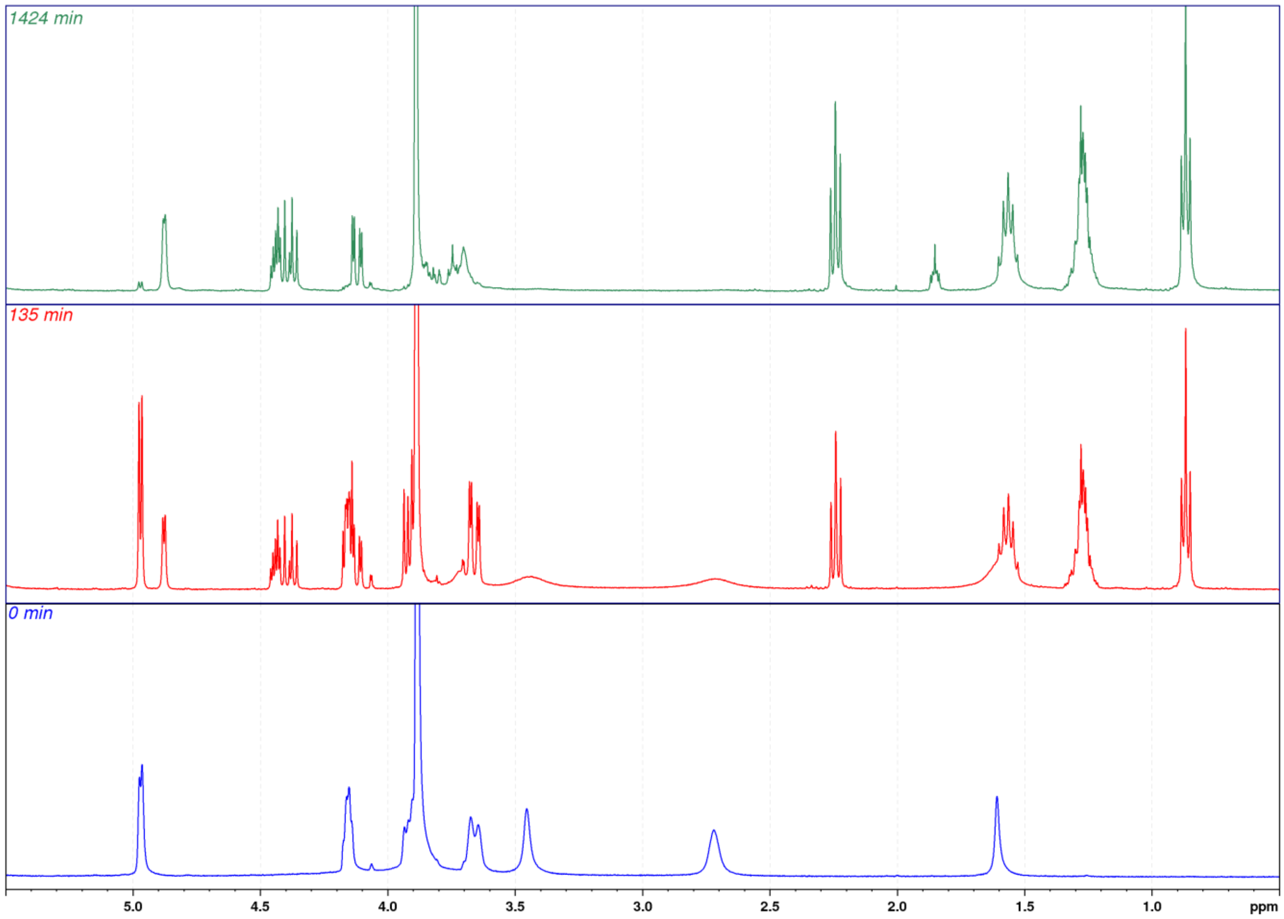

Supplementary Figure 8. Kinetic of guaiacylglycerol-β-guaiacyl ether enzymatic hexanoylation followed by ^{1}H NMR at at t= 0 min (in blue), after 135 min (in red) and after 24 h (in green) of biocatalysed hexanoylation; aliquots of 500 µL were periodically collected, dried under vacuum and dissolved in $CDCl_3$; spectra were calibrated on $CDCl_3$ at 7.26 ppm; zoom on 5.5-0.5 ppm; %Hexanoylation = Area(4.88 ppm)/(Area(4.98 ppm) + (Area(4.88 ppm)).

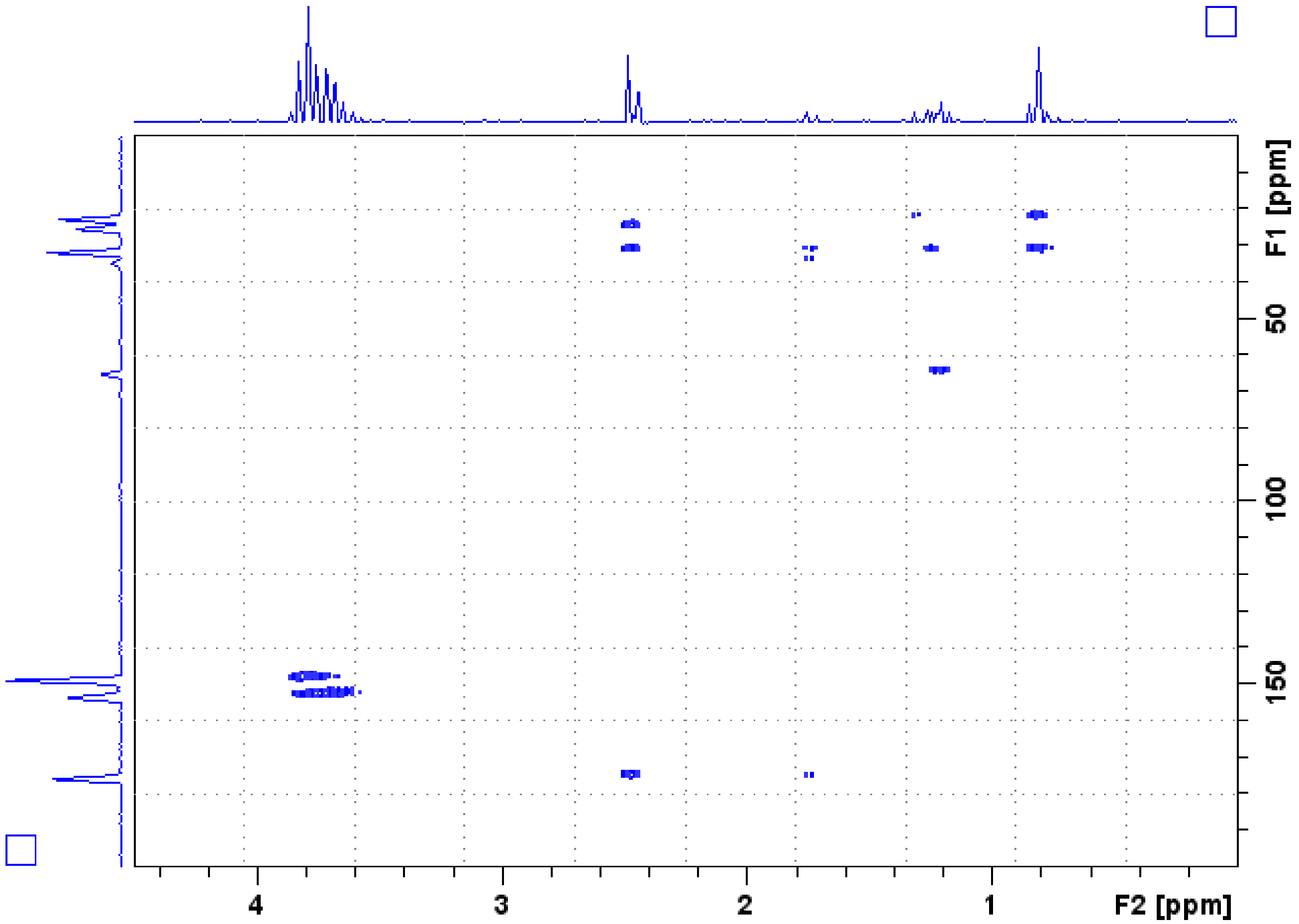

Supplementary Figure 9. ^{1}H-^{13}C HMBC NMR spectra in pyridine-d5 of DHPs enzymatically hexanoylated; zoom on 4.5-0/200-0 ppm.

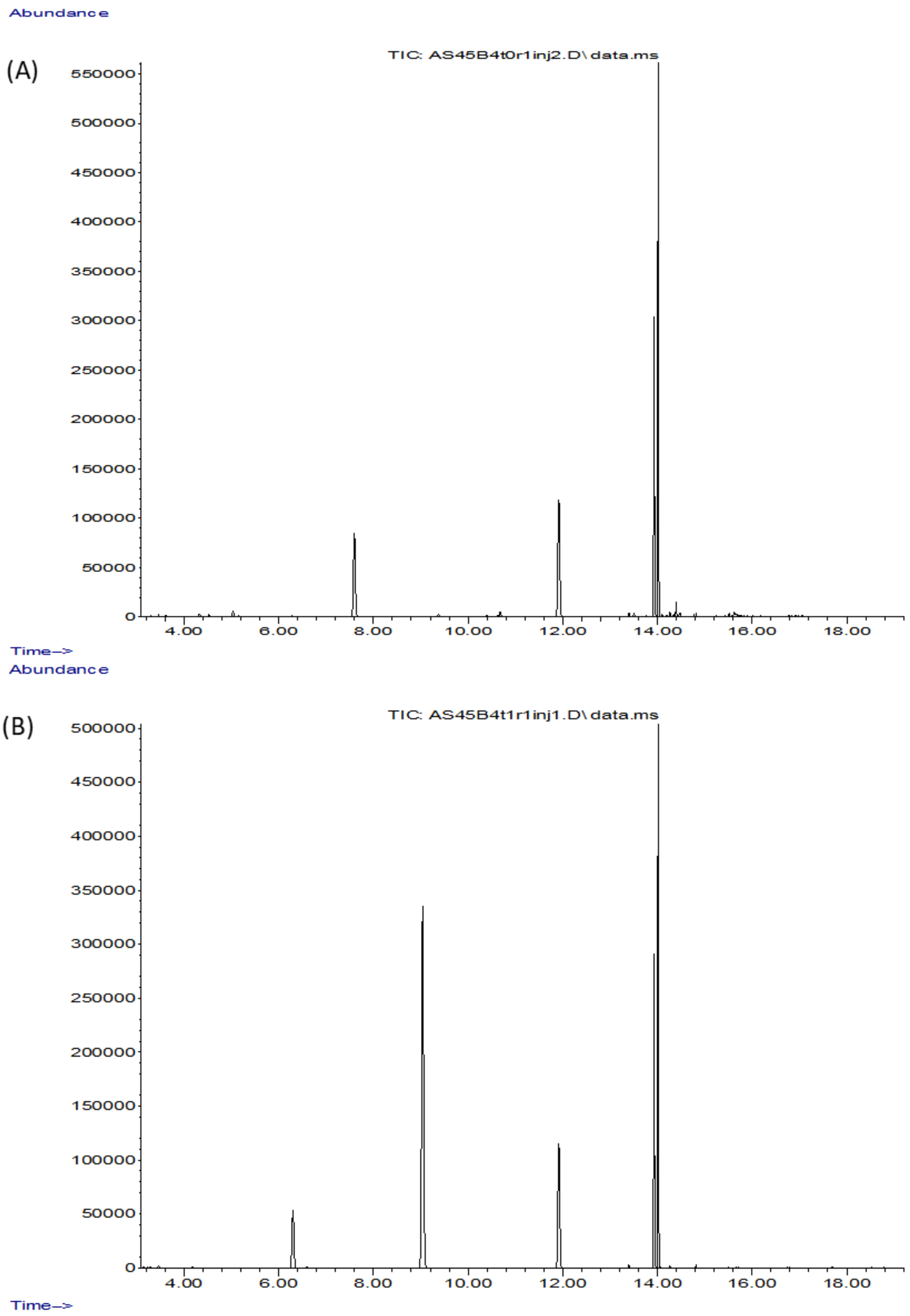

Supplementary Figure 10. GC-MS chromatograms of hexanoylated DHPs (A) for quantification of residual ethyl hexanoate and (B) for quantification of methyl hexanoate released by transmethylation; retention times are: Methyl pentanoate 6.30 min, Ethyl pentanoate 7.60 min, Methyl hexanoate 9.03 min, Ethyl hexanoate 10.40 min, Methyl heptanoate 11.92 min, Methyl octanoate 13.92 min and Tetradecane 14.03 min.

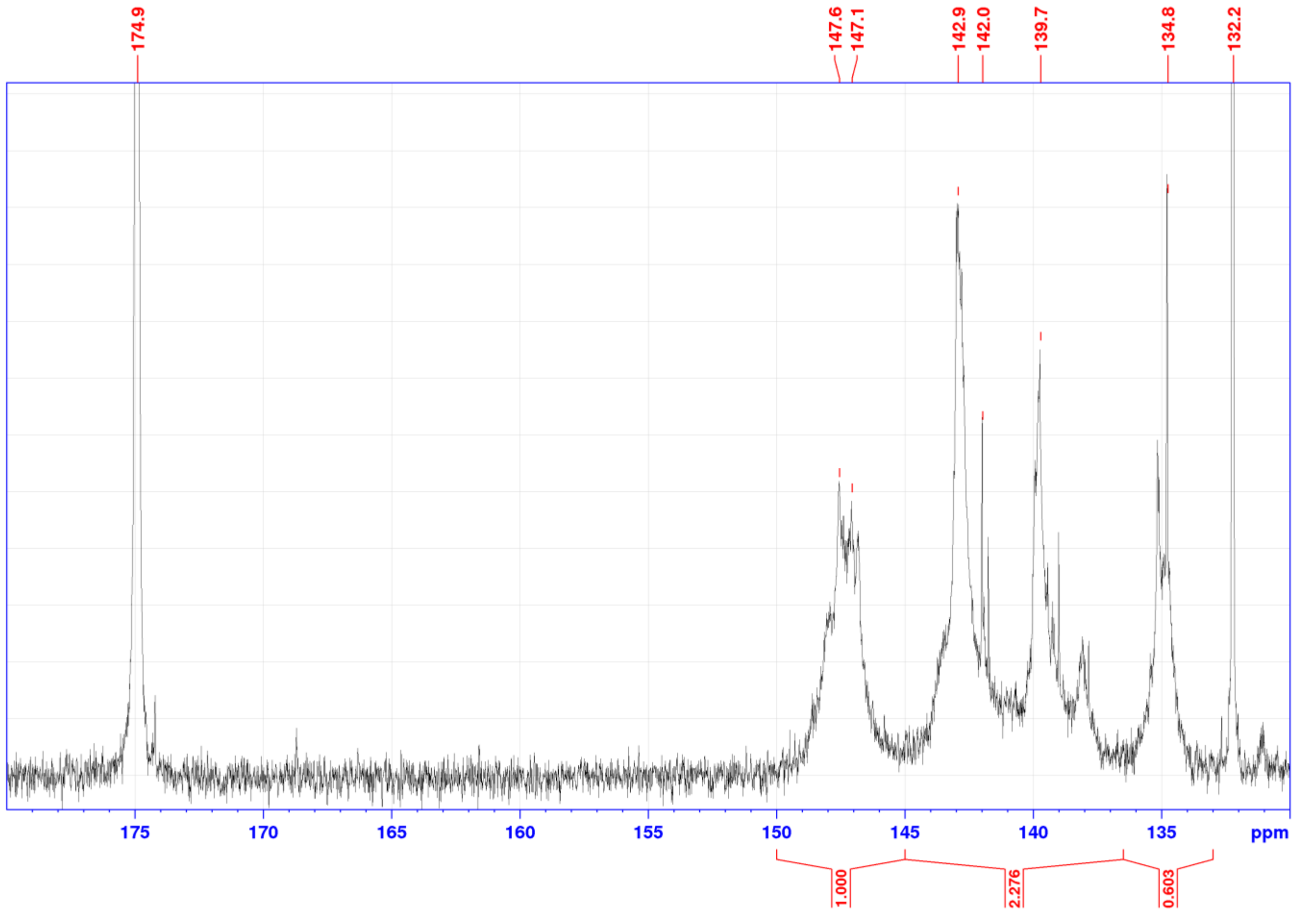

Supplementary Figure 11. ^{31}P NMR spectrum of technical soda lignins PB1000 after phosphorylation in pyridine/$CDCl_3$; spectrum calibrated on hydrolyzed phosphorylating reagent at 132.2 ppm; zoom on 180-130 ppm.

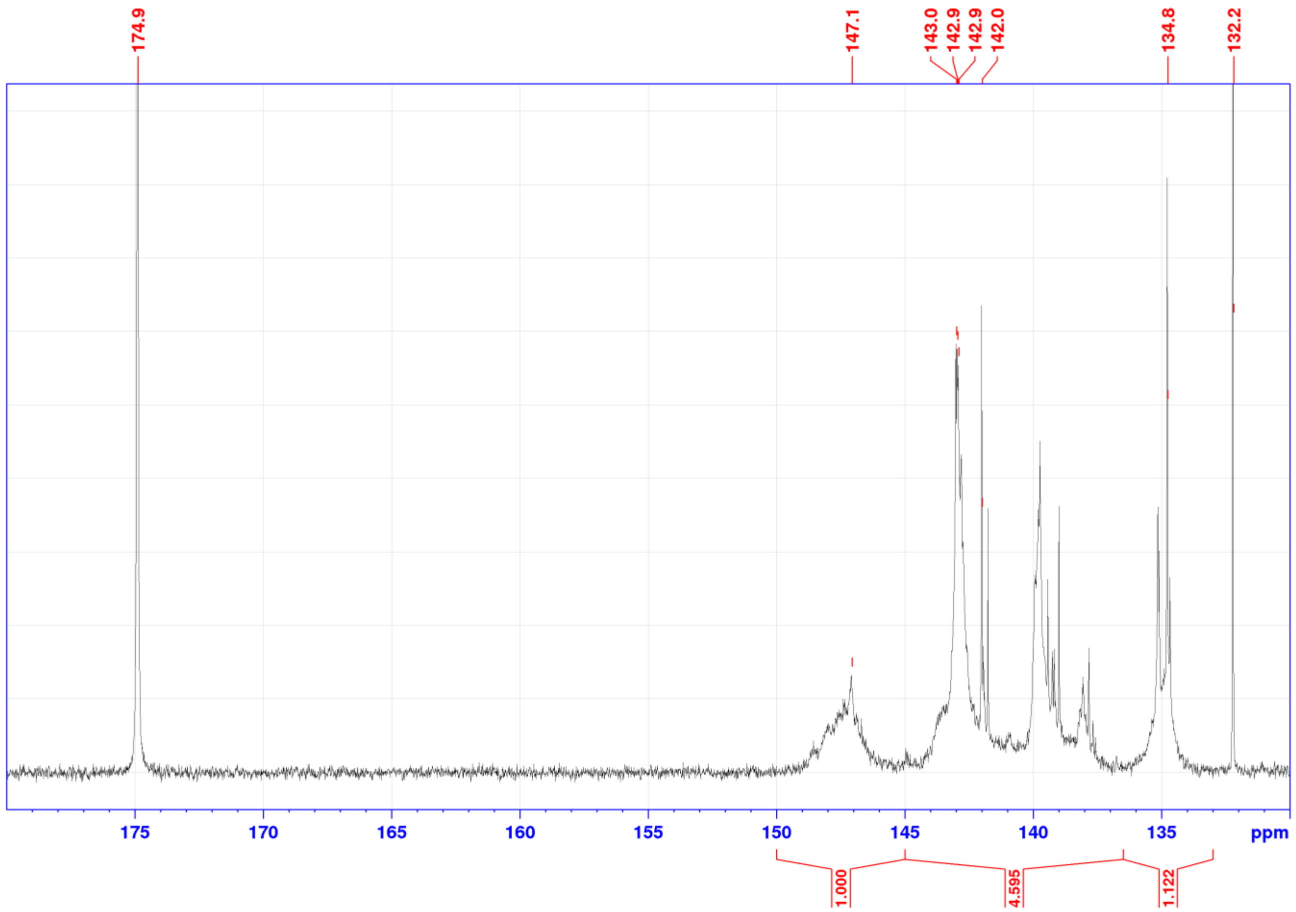

Supplementary Figure 12. ^{31}P NMR spectrum of F1 (ethyl acetate soluble) after phosphorylation in pyridine/$CDCl_3$; spectrum calibrated on hydrolyzed phosphorylating reagent at 132.2 ppm; zoom on 180-130 ppm.

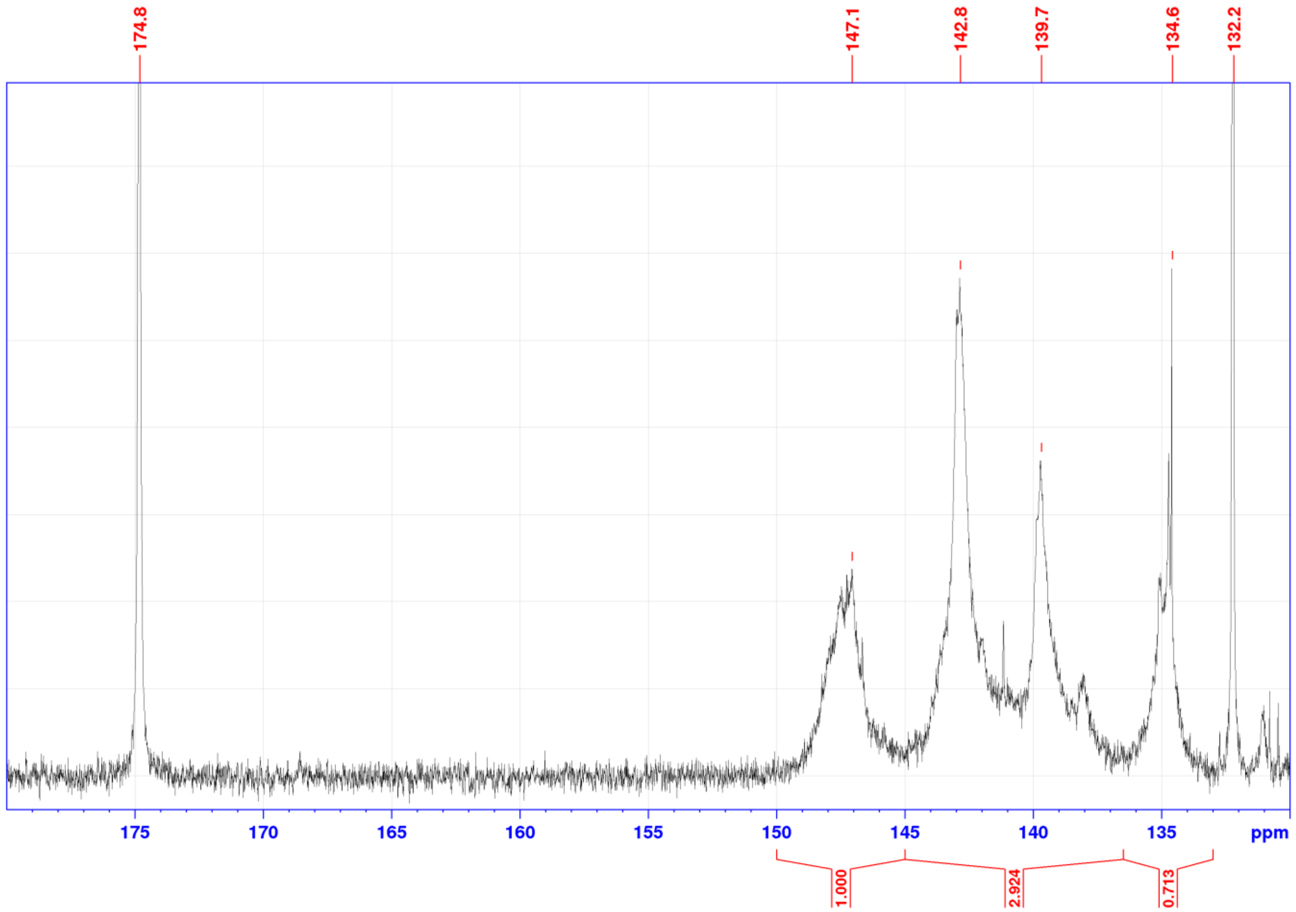

Supplementary Figure 13. ^{31}P NMR spectrum of F2 (ethyl acetate insoluble, MEK soluble) after phosphorylation in pyridine/$CDCl_3$; spectrum calibrated on hydrolyzed phosphorylating reagent at 132.2 ppm; zoom on 180-130 ppm.

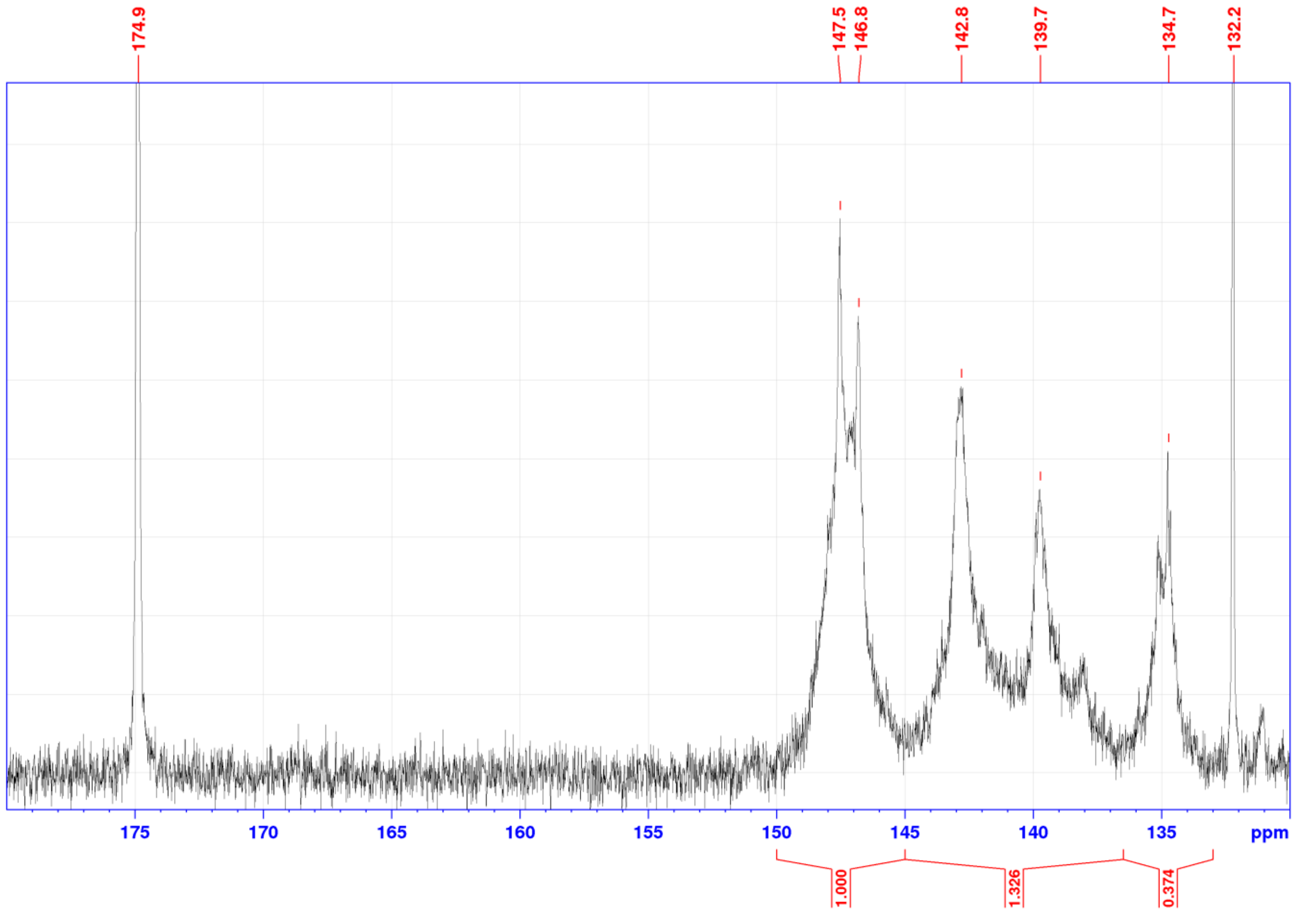

Supplementary Figure 14. ^{31}P NMR spectrum of F3 (MEK insoluble residue) after phosphorylation in pyridine/$CDCl_3$; spectrum calibrated on hydrolyzed phosphorylating reagent at 132.2 ppm; zoom on 180-130 ppm.
